# Review on biological active metabolites of Astragali radix and its mechanisms in treating allergic respiratory diseases

**DOI:** 10.3389/fphar.2026.1801620

**Published:** 2026-06-03

**Authors:** Zeyun Wang, Jie Cui, Shaoyan Zhang, Yu Wang, Lei Qiu, Zifeng Ma, Huimin Shen, Zhenhui Lu, Cui Li

**Affiliations:** Institute of Respiratory Diseases, Longhua Hospital, Shanghai University of Traditional Chinese Medicine, Shanghai, China

**Keywords:** allergic respiratory diseases, anti-inflammatory activity, immunoregulation, astragali radix, astragalus biologically active metabolites, pharmacological mechanisms

## Abstract

The global incidence of allergic respiratory diseases (ARDs) is rising, imposing severe socioeconomic and public health burdens worldwide. Astragali Radix (AR), a well-recognized traditional Chinese medicinal botanical drug with proven efficacy in ARDs management, exerts its therapeutic effects primarily through three major classes of bioactive metabolites: polysaccharides (e.g., APS), saponins (e.g., AS-II/IV/VII), and flavonoids (e.g., quercetin, calycosin). Of these, AS-IV and APS are the most extensively investigated, exhibiting prominent anti-inflammatory, immunomodulatory, anti-airway remodeling, and anti-fibrotic activities. Based on a comprehensive literature search across multiple authoritative databases, this narrative review innovatively focuses on ARDs (allergic asthma, ARh, AC, HP) from the perspective of a unified disease spectrum, summarizing the pharmacodynamic mechanisms of these AR metabolites in ARDs--pecifically, targeting core pathogenic signaling pathways (e.g., NF-κB, MAPK, TGF-β1/Smad) and regulating immune homeostasis (e.g., inhibiting ILC2 activation, rebalancing Th1/Th2 polarization, and suppressing Th17/IL-17-mediated inflammation). By integrating AR’s pharmacodynamic effects with its molecular targets and correlating preclinical evidence with clinical data (including RCTs), this review establishes a comprehensive foundation for the clinical application of AR and the development of novel targeted therapeutics for ARDs.

## 1 Introduction

Allergic diseases, one of the six most prevalent chronic diseases identified by the World Health Organization, are triggered by the body’s aberrant immune response to allergens. In recent years, the incidence of allergic respiratory diseases (ARDs), including allergic asthma and allergic rhinitis (ARh), has risen globally (Wang P. et al., 2023; Zhang et al., 2022) due to urbanization, industrialization, exposure to air pollution (Agache et al., 2022; To et al., 2020), climate change such as exacerbated pollen seasons (Anderegg et al., 2021; D'Amato et al., 2015), and long-term complications of coronavirus disease 2019 (Wechsler et al., 2022). The World Organization of Allergy White Paper has estimated that there are 300 million people with allergic asthma and 400 million with ARh worldwide. The global prevalence of chronic cough is approximately 10% (Song et al., 2015), among which atopic cough (AC) is recognized as one of the most common pulmonary etiologies (Côté et al., 2020; Lai et al., 2013). Additionally, hypersensitivity pneumonitis (HP) is an immune-mediated interstitial lung disease triggered by repeated inhalation of environmental or occupational antigens, with a prevalence of 0.3–0.9 per 100,000 population (Fernández Pérez et al., 2018; Raghu et al., 2020).

Global clinical guidelines for managing ARDs (e.g., asthma, ARh) recommend a multimodal therapeutic armamentarium, including: (1) anti-inflammatory agents [inhaled corticosteroids (ICS), intranasal corticosteroids (INCS), oral corticosteroids (OCS)]; (2) bronchodilators/decongestants [β2-agonists (short-acting: SABA; long-acting: LABA), anticholinergic drugs, oral/intranasal decongestants, theophylline]; (3) immunomodulators [leukotriene receptor modulators (LTRMs), antihistamines, allergen immunotherapy (AIT)]; (4) targeted biologics [e.g., anti-IgE, anti-IL-5/IL-5R, anti-IL-4R monoclonal antibodies]; and (5) adjunctive agents [sodium cromoglycate, primarily used for adjunctive symptom relief in refractory cases] (Global Initiative for Asthma [GINA], 2025; Chinese Thoracic Society, Chinese Medical Association et al., 2025).

However, current pharmacotherapeutic strategies still have two critical limitations: first, a high relapse rate (40%–60% within 3–6 months) is observed following drug withdrawal, particularly in patients with moderate-to-severe ARDs, which is attributed to the incomplete resolution of airway inflammation and immune dysregulation (See, 2023; Zhang J. et al., 2024); second, dose- and duration-dependent adverse events with long-term use: ICS/INCS are associated with local side effects (oropharyngeal candidiasis, hoarseness, nasal dryness; incidence 15%–25%), while OCS increase risks of systemic complications (dysglycemia, osteoporosis, adrenal insufficiency, and infections; relative risk 1.8–2.5) (GINA, 2024; Zdziarski et al., 2022). Furthermore, leukotriene receptor modulators (e.g., Montelukast) have been reported to exert adverse effects on neurological and psychiatric health (GINA, 2024); in addition, high doses of salbutamol may induce direct or indirect cardiovascular sequelae including tachycardia, electrocardiographic changes, as well as hypokalaemia and hypomagnesaemia (Lipworth, 2001). Additionally, long-term use of theophylline carries a narrow therapeutic window, often leading to gastrointestinal symptoms (nausea, vomiting) or cardiac arrhythmias (incidence ∼8%) in elderly patients (Zhang J. et al., 2024)

Traditional Chinese medicine (TCM) is a well-established and effective medical system with a history spanning several thousand years, among which botanical therapy serves as the predominant treatment modality (Chan and Ng, 2020). From the TCM perspective, the pathogenesis of ARDs is primarily associated with lung-spleen qi deficiency, defensive exterior instability, and dampness-phlegm retention. Specifically, the lung governs qi and regulates the defensive exterior, while the spleen acts as the source of qi and blood production; lung-spleen qi deficiency impairs the ability to secure the defensive exterior, rendering the respiratory tract vulnerable to invasion by external pathogenic factors (e.g., wind, cold, dampness) and thereby inducing symptoms such as sneezing, rhinorrhea, and asthma. Meanwhile, qi deficiency hinders the transportation of body fluids, leading to dampness accumulation and phlegm formation, which obstruct the airway and further exacerbate respiratory manifestations.

**FIGURE 1:**
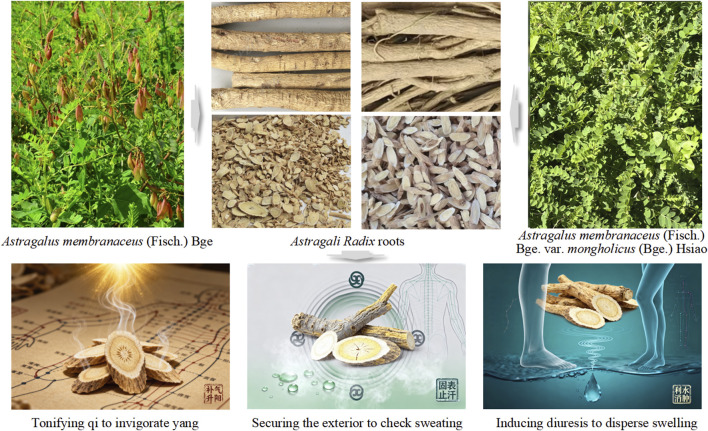
AR and its TCM effects. This figure displays the botanical sources of AR (*Astragalus membranaceus* (Fisch.) Bge and *Astragalus membranaceus* (Fisch.) Bge. var. mongholicus (Bge.) Hsiao), processed AR roots/slices, and its core TCM therapeutic effects: tonifying qi to invigorate yang, securing the exterior to check sweating, and inducing diuresis to disperse swelling—all of which provide a traditional pharmacological basis for AR’s application in ARDs.

Astragali Radix (AR, Huangqi), the dried root of *Astragalus membranaceus* (Fisch.) Bge. var. mongholicus (Bge.) Hsiao or *Astragalus membranaceus* (Fisch.) Bge. (Fabaceae), is recorded in the Pharmacopoeia of the People’s Republic of China as a classic TCM botanical drug with a sweet flavor, exerting the effects of tonifying qi to invigorate yang, securing the exterior to check sweating, and inducing diuresis to disperse swelling (Figure 1). The taxonomic identity of this plant species has been validated in accordance with the Medicinal Plant Name Service (MPNS, http://mpns.kew.org/mpns-portal/), with the full scientific name as *Astragalus mongholicus* Bunge [Fabaceae].

Clinically, AR is widely utilized in the management of ARDs, with accumulating evidence supporting its efficacy in allergic asthma (D'Avino et al., 2023; Qin et al., 2019), ARh (Hua et al., 2024; Li et al., 2021), AC (Wang et al., 2022; Molitorisova et al., 2021), and HP (Yan et al., 2020; Chen et al., 2010; Dong et al., 2018). In TCM Botanical formulas for ARDs, AR plays a pivotal role: it often serves as the sovereign botanical drug (the core metabolite targeting the pathogenesis, e.g., qi deficiency-induced immune dysregulation) and ranks as the second most frequently used botanical drug in formulations for ARh (Li et al., 2021), which reflecting its efficacy in reinforcing lung qi and stabilizing the defensive exterior to alleviate nasal symptoms (e.g., sneezing, rhinorrhea). For allergic asthma, AR maintains the fourth most commonly used botanical drug and the second most frequently combined botanical drug in asthma-specific formulas (Qin et al., 2019), where it synergizes with other botanical drugs (e.g., Ephedrae Herba, Armeniacae Semen Amarum) to enhance efficacy.

The pharmacological effects of AR on ARDs are mediated by its diverse chemical metabolites, including astragalus polysaccharides (APS), saponins, and flavonoids (Liu et al., 2024; Yao et al., 2024; Zheng et al., 2020). These show anti-inflammatory (Chen et al., 2023), anti-airway hyperresponsiveness (AHR) (Jin et al., 2017), anti-airway remodeling (Du et al., 2008), anti-oxidative damage (Wang et al., 2020), and anti-fibrotic effects (Zhang et al., 2020) by nuclear factor-kappa B (NF-κB), mitogen-activated protein kinase (MAPK), transforming growth factor-β1 (TGF-β1)/Smads, nuclear factor-E2-related factor 2 (Nrf2)/antioxidant response element (ARE), Nod-like receptor family pyrin domain-containing protein 3 (NLRP3)/Caspase1, phosphatidylinositol three kinase/protein kinase B (PI3K/AKT), JAK/STAT and Calcineurin/nuclear factor of activated T cells 4 (NFATc4) signaling pathway, and Th1/Th2 balance regulation, Th17/IL-17 inhibition, ILC2 function regulation immune homeostasis pathways.

Although several valuable reviews have elaborated on the pharmacological roles of AR in allergic or respiratory disorders (Bival Štefan, 2024; Zhang S. et al., 2025; Yuan et al., 2023; Tan et al., 2023), the present study provides a distinct and integrated perspective focused on ARDs as a unified disease spectrum. Through comprehensive literature searching and critical evaluation, this review offers several unique contributions: first, it provides a targeted analysis of AR’s immunomodulatory mechanisms specifically within the context of ARDs, rather than general allergic or respiratory conditions. Second, it achieves comprehensive integration of AR and its multiple classes of bioactive metabolites—including polysaccharides, saponins, and flavonoids—across the full spectrum of ARDs, including asthma, allergic rhinitis, allergic cough, and hypersensitivity pneumonitis. Third, it highlights emerging immune regulatory pathways that are central to ARDs pathogenesis, such as ILC2 regulation and NLRP3 inflammasome-mediated pyroptosis, thereby complementing the established focus on classical signaling axes. Fourth, it systematically connects preclinical mechanistic evidence with key translational considerations, including metabolite purity, pharmacokinetic properties, and delivery strategies relevant to clinical development. Fifth, it summarizes clinical evidence for both AR-containing metabolite formulations and single-metabolite preparations, supporting more targeted therapeutic development in ARDs.

Accordingly, the objectives of this review are threefold: first, systematically clarify the immunological mechanisms and molecular signaling networks of AR metabolites across the full spectrum of ARDs; second, highlight newly recognized regulatory functions of AR metabolites in understudied pathogenic pathways; and third, construct a translational framework to bridge mechanistic understanding with clinical application, thereby fostering the development of AR-based interventions for ARDs. This review not only focus on individual ARDs or single AR metabolites, instead, it innovatively integrates AR’s bioactive metabolites (especially polysaccharides) with the unified ARDs spectrum, providing a novel perspective for understanding the ethnopharmacological value of *Astragalus mongholicus* Bunge in treating ARDs.

## 2 Literature search and study selection

A narrative review approach was adopted to retrieve literature from PubMed, Web of Science, CNKI, WanFang Data and the Chinese Medical Journal Full-text Database, with no publication time restrictions. The search terms covered three core dimensions: AR and its bioactive metabolites, ARDs and related subtypes, as well as disease-relevant pharmacology, immune mechanisms, signaling pathways, toxicological features and clinical evidence. Retrieved studies were rigorously screened; methodologically sound publications were included, while non-peer-reviewed, low-quality and duplicate literature was excluded to guarantee the comprehensiveness and reliability of this review.

## 3 Biologically active metabolites of AR

The phytochemical complexity of AR enables multi-target engagement, pathway modulation, and distinctive clinical efficacy in ARDs therapy. To date, AR has revealed a diverse repertoire of 404 documented metabolites, mainly including APS, saponins, flavonoids, phenylpropanoids, alkaloids, and others (Liu et al., 2024). Among them, APS, saponins, and flavonoids metabolites are largely responsible for the pharmacological effects of AR (Liu et al., 2024; Yao et al., 2024; Zheng et al., 2020).

Although *Astragalus mongholicus* Bunge [Fabaceae] is a well-characterized botanical drug with a long history of use in TCM, this review provides a refined and mechanism-driven update to its ethnopharmacological profile in the context of allergic respiratory diseases (ARDs). Unlike previous broad summaries, this analysis specifically dissects the structure-activity relationship (SAR) of its bioactive polysaccharide fraction, which is recognized as a major contributor to anti-allergic efficacy. We systematically summarize the critical structural characteristics that determine pharmacological activity, including molecular weight distribution, monosaccharide composition (e.g., high content of galacturonic acid and arabinose), glycosidic linkage patterns, and the presence of protein moieties in heteropolysaccharides. This review establishes a clear link between these structural features and specific immunomodulatory effects in ARDs: for instance, low-molecular-weight homogeneous polysaccharides often exhibit superior bioavailability and targeting of ILC2s, while acidic polysaccharides with complex branching structures are potent inhibitors of the NLRP3 inflammasome. By integrating structural biology with pharmacology, this summary advances our understanding from general efficacy to predictive structural rules, providing a rational basis for the development of standardized polysaccharide preparations with optimized anti-ARD activity.

### 3.1 APS

Most of APS are heteropolysaccharides (e.g., APS I, II, III, AMon-S *etc.*) with different ratio of multiple monosaccharide units, such as glucose, glucuronic acid, arabinose, rhamnose, fructose, mannose, galactose, galacturonic acid and so on (Jin et al., 2014). Some homogeneous polysaccharides have also been successfully isolated and characterized from APS, such as APS-D1, which contains →4)α-D-Glcp-(1→ residue main chain with →3)-β-D-Glcp-(1→ residue and terminal-α/β-D-Glcp-(1→ side chain (Long et al., 2024), APS-A1 (mainly includes T-Glcp, 1,4-Glcp and 1,4,6-Glcp) and APS-B1 (mainly includes 1,5-Araf, T-Glcp, 1,4-Glcp, 1,6-Galp and 1,4,6-Glcp) (Chen et al., 2023). Studies have shown that the immunomodulatory mechanism of APS is mainly related to the content of mannose, glucose, xylose and fucose. But due to the difficulty of completely separating the metabolites of APS, the current experimental researches mostly are about APS (Jin et al., 2014), with immunomodulatory, blood lipids and glucose regulating, radiation protection, anti-aging, anti-tumor, antiviral, anti-fibrosis and bacteriostasis (Zheng et al., 2020) effects. During allergic respiratory disease attacks, APS mainly plays a role of immunoregulation, anti-oxidation, anti-inflammation and anti-fibrosis effects—targeting the multi-pathological processes of ARDs.

### 3.2 Saponins

More than 117 saponins with structural types including cycloartane, oleanolic acid, and high oxygenated malabaricane, have been isolated from AR (Liu et al., 2024) and named astragalus saponins. Cycloastragenol is used as a glycoside to form monosaccharide or disaccharide chains through glycosylation, with glucose, xylose, or xylose as the main glycosides. Astragaloside I-VII, Isoastragaloside I-III, Soyasaponin I and cyclocephaloside II are the core types of astragalus saponins. Among them, astragaloside IV (AS-IV) is the qualitative and quantitative index of AR. The *Pharmacopoeia of the People’s Republic of China* (ChP) officially specifies that the content of astragaloside IV (C_41_H_68_O_14_) should not be less than 0.010%/g (dry product). Astragalus saponins possesses multiple pharmacological activities in immunomodulatory, blood glucose regulating, hepatoprotection, anti-nerve injury, anti-cardiovascular activities, antitumor, antiviral, anti-inflammation, anti-oxidation, anti-fibrosis and bacteriostasis activities (Dong et al., 2023). During allergic respiratory disease attacks, astragalus saponins primarily function through immunomodulation, anti-inflammatory activity and anti-fibrosis effects.

### 3.3 Flavonoids

There are 160 flavonoids contented in AR (Liu et al., 2024), the core is the flavonoid skeleton (such as A-ring hydroxylation and B-ring methoxy substitution), and some are formed into glycosides through glycosylation (such as flavonol glycosides). Among these bioactive metabolites, calycosin serves as a key qualitative and quantitative marker for AR quality evaluation. Specifically, the ChP officially specifies that the content of calycosin (C_22_H_22_O_10_) in AR shall not be less than 0.020%/g on a dry basis. Other representative metabolites such as formononetin (aglycone) and calycosin (hydroxy substituted derivatives) have structural differences that affect their solubility and bioavailability. Flavonoids develop various pharmacological effects such as regulating the immune function, anti-inflammation, anti-oxidation, anti-fibrosis and bacteriostasis in the treatment of ARDs (Dong et al., 2023).

**FIGURE 2:**
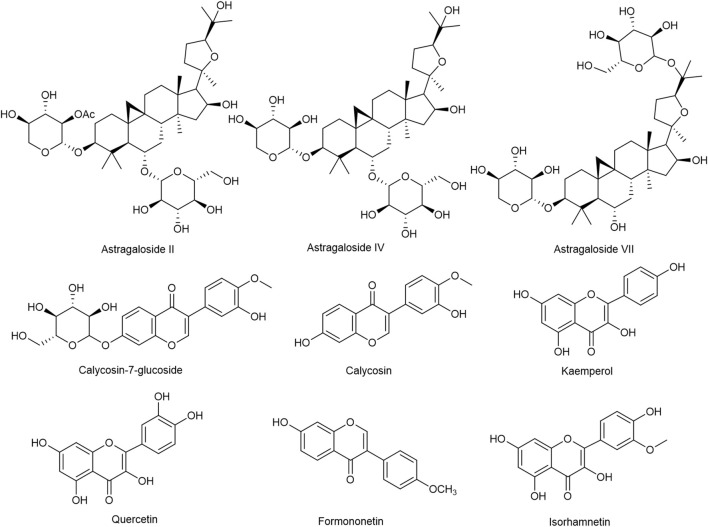
Chemical structures of major bioactive metabolites from AR. This figure presents the chemical structures of key saponins (AS-II, AS-IV, AS-VII) and flavonoids (calycosin-7-glucoside, calycosin, kaempferol, quercetin, formononetin, isorhamnetin) isolated from AR, which are the primary active metabolites responsible for anti-inflammatory and immunomodulatory effects in ARDs. Abbreviations: AR, Astragalus radix; AS-II, astragaloside II; AS-IV, astragaloside IV; AS-VII, astragaloside VII.

The review of the current relevant literature has shown that studies on the ARDs’ regulative properties of AR mainly focused on APS, AS-II, AS-IV, AS-VII, calycosin-7-glucoside, calycosin, kaempferol, quercetin, formononetin and isorhamnetin. The chemical structures of these metabolites are shown in Figure 2.

## 4 Pharmacodynamics of AR and its metabolites in allergic ARDs

AR has attracted the attention of both domestic and foreign experts and scholars owing to its rich chemical composition and strong pharmacological effects (Shi et al., 2024). AR and its active metabolites have significant therapeutic effects on ARDs, including allergic asthma, ARh, AC and HP. The pharmacodynamics of AR and its metabolites in ARDs have been reviewed.

### 4.1 Pharmacodynamics of AR’s metabolites in allergic asthma

Bronchial asthma is a heterogeneous airway disease characterized by chronic inflammation and variable expiratory airflow limitation (GINA, 2024). As a progressive pathological process, prolonged disease duration drives airway remodeling, which further leads to structural airway damage—an established strong prognostic indicator for disease progression and therapeutic response (Savin et al., 2023). Allergic asthma is the most prevalent phenotypic subtype, accounting for 40%–50% of adult cases and approximately 80% of pediatric cases (nese Thoracic Society Chinese Medical Association et al., 2025). Its pathogenesis is closely linked to key pathological features including goblet cell proliferation, smooth muscle cell hyperplasia, excessive mucus secretion, AHR, airway remodeling, and exacerbated oxidative stress (Yuan et al., 2020) —all of which contribute to the development of structural airway damage and poor clinical outcomes.

#### 4.1.1 Effects of APS

Wang et al. (Wang et al., 2024) demonstrated that intraperitoneal (i.p.) injection of APS (150, 200, 300 mg/kg) in a rat model of allergic asthma restored Th1/Th2 balance, alleviated lung pathological damage (including goblet cell and smooth muscle cell hyperplasia), and reduced pro-inflammatory cytokine secretion. This effect was mediated by inhibiting aberrant TGF-β1/Smads signaling—a pathway that drives immune dysregulation, progressive airway remodeling, and irreversible lung fibrosis, and serves as a strong prognostic indicator for disease severity and long-term outcomes. By suppressing TGF-β1/Smads-mediated airway remodeling, APS not only mitigates acute inflammatory injury but also potentially improves clinical prognosis by intervening in progressive airway structural deterioration, which is closely linked to treatment response in allergic asthma (Savin et al., 2023) (Table 1). Zhang and Ma discovered that APS (100 mg/kg, i.p.) induced protective immunity against allergic asthma by regulating the function of dendritic cells (DCs), natural killer (NK) cells, and regulatory T cells (Treg cells). It corrected Th1/Th2 imbalance by promoting Th1 polarization (e.g., increasing IFN-γ secretion) and enhancing Treg-mediated immune tolerance, thereby suppressing excessive type 2 inflammation (Zhang and Ma, 2020) (Table 1). Besides, previous studies showed that maintaining a certain amount or certain species of gut microbial communities plays an indispensable role in the effects of regulatory T-cells on preventing hypersensitivity (Wang F. et al., 2023; Luo et al., 2018; Loo et al., 2014). Zhang and his members demonstrated that in ovalbumin (OVA)-induced asthmatic mice, intragastric (i.g.) administration of APS (5, 10 g/kg) reduced abundance of Firmicutes, increased the abundance of Bacteroidete, and corrected the gut microbial imbalance associated with asthma, which thereby alleviated airway inflammation and EOS infiltration caused by asthma (Zhang J. et al., 2024) (Table 1). These mechanisms regulation highlights APS as a promising candidate for targeting systemic immune dysregulation in allergic asthma.

**TABLE 1:** Mechanisms of APS in the treatment of ARDs.

Metabolites	Research subjects	Dosage	Results
APS	OVA-induced asthmatic mice	100 mg/kg (i.p.) for 14 days	Lower airway resistance; DCs↓; anti-infammatory NK cells (considered to be regulators of Th1 or Th2 cytokine production) and Treg cells↑; IL-4 and IL-10↓ (Zhang and Ma, 2020)
APS	OVA-induced asthmatic mice	5, 10 g/kg (i.g.) for 7 days	EOS↓; regulate the gut microbiota and the metabolites, thus improve airway inflammation and AHR (Zhang et al., 2024b)
APS	OVA-induced asthmatic rats	150, 200 and 300 mg/kg (i.p.) for 14 days	Reduce the damaged area of bronchial mucosa, smooth muscle thickness, and changes in lung tissue morphology; inhibit airway remodeling; IFN-γ↑, IL-4↓, TGF-β1, Smad2, Smad3↓; regulate Th1/Th2 cell balance; inhabit TGF-β1/Smads pathways (Wang et al., 2024)
APS	OVA-induced allergic rhinitis rats	5, 10 and 15 mg/kg (i.n.) for 10 days	Reduce EOS infiltration and the expression of IL-4 in the nasal mucosa tissue (Wang et al., 2020)
APS	OVA-induced allergic rhinitis guinea pigs	25, 50, and 100 mg/kg (i.g.) for 10 days	Suppress OVA-sIgE, TNF-α, Foxp3, IL-17, TGF-β, and IL-6 levels; increase CD25+Foxp3+Treg cell proportion; reduce CD4+IL17+Th17 cell proportion; block NF-κB p65 and downregulate NF-κB expression; reduced Treg/Th17 imbalance (He et al., 2022)
APS	BLM-induced IPF model mice	25, 50, and 100 mg/kg (i.g.) for 28 days	Inhibit collagen deposition; IL-6, IL-1β, and TNF-α↓; TLR4, p-p65/p65↓and IκBα↑; BLM↓and Bcl-2↑; inhibit TLR4/NF-κB signaling pathway (Wei et al., 2023)
APS	BLM-inducted PF mice; human lung epithelial A549 cells	200 mg/kg (i.g.) for 28 days 50 mg/mL for 3 days	Alleviate the BLM-induced upregulation of collagen and fibronectin; reverse TGF-β1-induced EMT and NF-κB pathway activation, along with a decrease in vimentin and α-SMA expression and an increase in the E-cadherin expression (Zhang et al., 2020)
APS-A1, APS-B1	LPS-induced RAW264.7 cells	12.5, 25, 50, 100, and 200 μg/mL for 24 h	IL-1β, iNOS and TNF-α↓; inhibit the phosphorylation; levels of IκB and p65 proteins inhibit NF-κB pathways (Chen et al., 2023)

Table 1 summarizes key preclinical studies on the therapeutic effects and underlying mechanisms of APS *in vivo* and *in vitro* trials of ARDs, including OVA-induced asthma/ARh, BLM-induced pulmonary fibrosis, and LPS-induced inflammation models. It details dosage regimens, key cellular and molecular outcomes related to inflammation, immune regulation, airway remodeling, and fibrosis, with corresponding references.

Abbreviations: APS, Astragalus polysaccharides; ARDs, allergic respiratory diseases; ARh, Allergic Rhinitis; OVA, ovalbumin; i.p., intraperitoneal; i.g., intragastric; in., intranasal; DCs, dendritic cells; NK cells, natural killer cells; Treg cells, regulatory T cells; IL, interleukin; EOS, eosinophils; AHR, airway hyperresponsiveness; IFN, interferon; TGF-β, transforming growth factor-β; Th, T helper; Treg, regulatory T cell; BLM, bleomycin; IPF, idiopathic pulmonary fibrosis; PF, pulmonary fibrosis; TLR4, Toll-like receptor 4; NF-κB, nuclear factor-κB; IκBα, inhibitor of nuclear factor-κB α; Bcl-2, B-cell lymphoma 2; APS-A1/APS-B1, homogeneous APS fractions; LPS, lipopolysaccharide; iNOS, inducible nitric oxide synthase; TNF-α, tumor necrosis factor-α.

#### 4.1.2 Effects of saponins

Astragalosides is the major class of triterpenoid saponins in AR (e.g., AS-II, AS-IV, AS-VII, Macrophyllosaponin B [Mac B]), exhibiting prominent anti-asthmatic effects through multi-targeted regulation of inflammation, immune balance, and airway remodeling--a key pathological process linked to disease prognosis. Wu found that in OVA-induced asthmatic rats, intraperitoneal (i.p.) injection of Astragaloside II (AS-II) (300, 600, 900 mg/kg) dose-dependently reduced the counts of EOS, neutrophils, lymphocytes, and macrophages in bronchoalveolar lavage fluid (BALF). It also downregulated the levels of inflammatory factor IL-6, IL-21, and TGF-β1 in BALF, as well as the protein expression of IL-21 and phosphorylated STAT3 (p-STAT3) in lung tissues, thereby reducing polarization of alternatively activated M2 macrophages and fibroblasts, slowing down the elevated production of extracellular matrix (ECM) metabolites and the development of fibrosis, thus repairing lung function, and mitigating structural airway damage associated with poor prognosis (Savin et al., 2023; Wu et al., 2021) (Table 2). At the same time, Du and his members observed that in OVA-induced asthmatic mice, oral administration of Astragaloside IV (AS-IV) (50 mg/kg) significantly reduced EOS and goblet cell counts in BALF, along with BALF levels of IL-4, IL-5, and IL-13, and serum total immunoglobulin E (IgE) (Du et al., 2008) (Table 2). After that, he further revealed that AS-IV (50 mg/kg, i.g.) markedly inhibited airway inflammation and remodeling, which was attributed to its suppression of α-smooth muscle actin (α-SMA) and vascular endothelial growth factor (VEGF) expression (Du et al., 2011) (Table 2). Another doctor Zhang found that AS-IV (100 mg/kg, i.p.) alleviated lung inflammation in OVA-induced asthmatic mice by reversing the proliferative phenotype of platelet-derived growth factor-BB (PDGF-BB)-stimulated airway smooth muscle cells (ASMCs), thus improving airway structural integrity and potential prognosis (Zhang H. et al., 2024) (Table 2). In addition, Nalbantsoy discovered that Astragaloside VII (AS-VII) and Mac B modulated ARDs by promoting the secretion of type 1 helper T cell (Th1) cytokines, suppressing type 2 helper T cell (Th2) cytokines and type 2 inflammatory factors, and enhancing immune regulation (Nalbantsoy et al., 2012).

**TABLE 2:** Mechanisms of saponins from AR in the treatment of ARDs.

Metabolites	Research subjects	Dosage	Results
AS-II	OVA-induced asthmatic mice	0.3, 0.6 and 0.9 mg/kg (i.p.) for 7 days	Downregulate the numbers of EOS, neutrophils, lymphocytes, and macrophages in BALF, reduce the contents of IL-6, IL-21, and TGF-β1, and protein expression of IL-21 and p-STAT3 in lung tissues dose-dependently (Wu et al., 2021)
AS-IV	OVA-induced asthmatic mice	10, 20 and 40 mg/kg (i.g.) for 28 days	Suppress AHR; reduce IL-4, IL-5, and IL-17A levels and increase INF-γ levels in the BALF; inhibit phosphorylation of S6RP and p70S6K; inhibit mTORC1 signaling pathway (Jin et al., 2017)
AS-IV	OVA-induced asthmatic mice	50 mg/kg (i.g.) for 56 days	EOS↓; AHR↓; IL-4 and IL-13↓; TGF-β1↓; total IgE↓; reduce peribronchial TGF-β1 and α-SMA; inhibit airway remodeling, including subepithelial fibrosis, smooth muscle hypertrophy, and goblet cell hyperplasia (Du et al., 2008)
AS-IV	OVA-induced asthmatic mice	50 mg/kg (i.g.) for 56 days	Reduce airway resistance; suppress overexpression of TGF-β1, TSLP mRNA, IL-4 and IL-13 (Du et al., 2011)
AS-IV	OVA-induced asthmatic mice	20, 40 mg/kg (i.g.) for 30 days	Reduce airway resistance and inflammatory cell infiltration; reduce EOS and IL-4 levels; increase IFN-γ and IL-10 levels in BALF; increase Treg and regulate Th1/Th2 cytokine (Huang et al., 2014)
AS-IV	OVA-induced asthmatic mice PDGF-BB-induced ASMCs	100 mg/kg (i.p.) for 28 days 25, 50, 100 and 200 μM for 24 h	suppress aberrant proliferation and promote ASMCs pyroptosis; block HMGB1/RAGE axis to inactivate NF-κB pathway (Zhang et al., 2024a)
AS-IV	OVA-induced asthmatic mice	50, 100 mg/kg (i.g.) for 28 days	IL-4, IL-5, IL-13 and the counts of total leucocytes, neutrophils, lymphocytes and EOS↓; the protein expressions of p-JAK2 and p-STAT6; downregulate the activity of JAK2/STAT6 signaling pathway (Yang and Wang, 2019)
AS-IV	His-induced NECs	20, 40 and 60 µM for 30 min	inhibit inflammatory cytokines secretion of IL-6, IL-8, MCP-1, IL-1β, IL-18, p65, IL-1B, TNF-α, GM-CSF, eotaxin, and MUC5AC; downregulate expression levels of CXCL11, CXCL2, MUC5AC, CCL3, TNF, IL-1β, IL-18, and NF-κB (Guo and Xu, 2021)
AS-IV	OVA-induced ARh mice	25, 50 mg/kg (i.g.) for 21 days	Alleviate the inflammatory response, nasal symptoms and mucosa remodeling, and decrease the serum levels of OVA-specific IgE; T-box, T-bet, Foxp3↑; GATA-3, RORγt↓; mediate the Th1/Th2 cell balance (Li et al., 2017a)
AS-IV	OVA-induced ARh mice	12.5 25 and 50 mg/kg (i.g.) for 27 days	Th17 and EOS↓; Treg↑; IL-10↑,IL-6, IL-17, TgE and TGF-β1↓; IL-17 and TGF-βmRNA↓; promote Th17/Treg balance (Zhang and Tian, 2022)
AS-IV	BLM-induced pulmonary fibrosis rats; alveolar epithelial cells A549	20 mg/kg (i.g.) for 14 days 100 μg/mL for 48 h	Alleviate collagen deposition; the levels of MDA↓; the levels of SOD and GSH-PX↑; the release of cytokines TNF-α, IL-6 and α-SMA↓; the expression of E-cadherin and FOXO3a↑; reverse the progression of BLM-induced EMT (Qian et al., 2018)
AS-IV	BLM-induced pulmonary fibrosis rats	10, 20 and 50 mg/kg (i.p.) for 28 days	Inhibit oxidative stress, the levels of MDA, SOD, T-AOC, and ROS in lung tissue↓; reduce inflammatory response, the levels of TNF-α, IL-1β, and IL-6 in BALF↓ (Yu et al., 2016)
AS-IV	BLM-induced pulmonary fibrosis rats	10 mg/kg (i.g.) for 28 days	Reduce collagen secretion, lower type III collagen, hydroxyproline and -SMA in lung tissue, decrease LN, HA, and HMGB1 in serum, attenuate BLM-induced extracellular matrix deposition (Li et al., 2017b)

Table 2 summarizes preclinical evidence for the anti-ARDs effects of major astragalus saponins (e.g., AS-II, AS-IV), detailing their efficacy in OVA-induced asthma/ARh, BLM-induced pulmonary fibrosis, and other relevant models, along with key signaling pathways (e.g., JAK/STAT, NF-κB, TGF-β/Smad) and references.

Abbreviations: AR, Astragalus radix; ARDs, allergic respiratory diseases; ARh, Allergic Rhinitis; AS-II, astragaloside II; AS-IV, astragaloside IV; OVA, ovalbumin; i.p., intraperitoneal; i.g., intragastric; EOS, eosinophils; IL, interleukin; TNF-α, tumor necrosis factor-α; BALF, bronchoalveolar lavage fluid; AHR, airway hyperresponsiveness; TGF-β, transforming growth factor-β; α-SMA, α-smooth muscle actin; TSLP, thymic stromal lymphopoietin; PDGF-BB, platelet-derived growth factor-BB; ASMCs, airway smooth muscle cells; HMGB1, high mobility group box 1; RAGE, receptor for advanced glycation end products; NECs, nasal epithelial cells; MUC5AC, mucin 5AC; MDA, malondialdehyde; SOD, superoxide dismutase; GSH-Px, glutathione peroxidase; LN, laminin; HA, hyaluronic acid.

#### 4.1.3 Effects of flavonoids

Plenty of experiments were conducted on flavonoids, especially calycosin, quercetin, kaempferol and isorhamnetin. For calycosin, Tian found that intraperitoneal injection of calycosin (20, 40 mg/kg) in asthmatic mice inhibited ILC2 activation, attenuated macrophage M2 polarization, reduced counts of EOS, neutrophils, macrophages, and lymphocytes in BALF, and decreased the release of Th2-type cytokines (IL-4, IL-5 and IL-13), which in turn might alleviate airway remodeling and structural damage linked to prognosis (Tian et al., 2024) (Table 3). Zhang revealed that intragastric administration of formononetin (25 mg/kg) effectively inhibited the release of pro-inflammatory cytokines, thereby suppressing airway inflammation in house dust mite (HDM)-sensitized asthmatic mice (a model involving human bronchial epithelial cell activation) (Zhang et al., 2023) (Table 3). Yi discovered that in OVA-induced asthmatic mice, i.g. administration of formononetin (10, 20, 40 mg/kg) regulated Th2/Th17 cell balance by significantly reducing the proportion of Th17 cells and the secretion of Th17-related inflammatory factors (e.g., IL-17A), further alleviating airway inflammation (Yi et al., 2020) (Table 3). For quercetin, Gupta and his team found that intraperitoneal injection of quercetin (0.2, 1, 5, 25 mg/kg) inhibited mast cell activation, which contributed to its regulatory effects on OVA-induced asthmatic mice (Gupta et al., 2016) (Table 3). For Kaempferol, Xu demonstrated that oral administration of kaempferol (20 mg/kg) downregulated the levels of IL-4, IL-5, IL-13, IL-25, IL-33, Beclin-1, and nicotinamide adenine dinucleotide phosphate oxidase 4 (NOX4) in asthmatic models. This finding was also supported by *in vitro* experiments, where kaempferol (1, 5, 10, 20, 40 μg/mL) intervened in human bronchial epithelial cells (BEAS-2B)—confirming that kaempferol improves airway inflammation and remodeling by suppressing NOX4-mediated autophagy (Xu et al., 2023) (Table 3). Additionally, Gong found that kaempferol (1–20 μM *in vitro*; 10, 20 mg/kg orally *in vivo*) attenuated tumor necrosis factor-α (TNF-α)-induced expression of epithelial intercellular adhesion molecule-1 (ICAM-1) and monocyte chemoattractant protein-1 (MCP-1) at the transcriptional level—reducing EOS-epithelial cell interactions and airway inflammation in airway epithelial cells of allergic asthmatic mice (Gong et al., 2012) (Table 3). For isorhamnetin, Zhu discovered that in OVA-induced asthmatic mice, i.p. injection of isorhamnetin (50, 150 mg/kg) inhibited allergic airway inflammation by altering Th1/Th2 cell balance. It specifically suppressed Th2 cell-mediated immune responses (e.g., reducing IL-4/IL-13 secretion and IgE production), thereby mitigating asthma phenotypes (Zhu et al., 2021) (Table 3).

**TABLE 3:** Mechanisms of flavonoids from AR in the treatment of ARDs.

Metabolites	Research subjects	Dosage	Results
TFA	LPS-induced RAW 264.7 macrophage of mice	10, 25 and 100 μg/mL for 12 h	Inhibit iNOS and COX-2 protein levels; inhibit the phosphorylation of p38 and JNK and IKKα/β, IκBα and the expression NF-κB, p65, regulate MAPK and NF-κB signaling pathways (Li et al., 2018)
Quercetin	OVA-induced asthmatic mice	0.2, 1, 5 and 25 mg/kg (i.p.) for 3 days	Mast Cells↓; OVA-sIgE, IL-4, IL-5, PGD2, mMCPT-1 Cys-L and TSLP ↓; c-MAF, GATA-3, NFAT and SOCS-3↓; FcεR1, Syk, c-Yes,PI-3,p-PI-3,PLC-γ2,and p-PLC-γ2↓; promote Thl/Th2 cytokine balance (Gupta et al., 2016)
Quercetin	OVA-induced allergic rhinitis rats	80 mg/kg (i.p.) for 7 days	EOS↓; COX-2↓; reduce total and OVA-specific IgE values (Sagit et al., 2017)
Quercetin	OVA-induced allergic rhinitis mice	20, 35 and 50 mg/kg (i.g.) for 13 days	Th17, EOS and goblet cells↓ and the proportion of Treg↑; IgE, IgG1, histamine↓; L-4, IL-5, TGF-β, IL-6, TNF-α and IL-17↓, and IFN-γ↑; RORγt mRNA↓ and Foxp3 mRNA↑; regulate Th1/Th2 and Th17/Treg balance; downregulate the levels of COX-2, p-IkBα and nuclear-p65, suppress NF-κB pathway (Ke et al., 2023)
CG	LPS-induced RAW 264.7 cells	5, 12.5, 25, 50 and 100 μM for 2, 18 h	Inhibit the productions of NO, PGE2, TNF-α, IL-1β and IL-6; suppress mRNA expression of iNOS, COX-2 and protein phosphorylation of IκBα, p65, ERK, JNK, and p38, and inhibit the NF-κB and MAPK signal pathway (Dong et al., 2018)
Calycosin	OVA-induced asthmatic mice ILC2, RAW264.7 macrophages	20, 40 mg/kg (i.p.) for 6 days 5, 10 μM for 48 h	EOS, neutrophils, macrophages and lymphocytes↓; IL-4, IL-5, IL-13, IL-33, ARG1, IL-10, YM1, MRC1 and ST2↓ (Tian et al., 2024)
Calycosin	BLM-induced pulmonary fibrosis mice MLE-12 cells	7, 14 mg/kg (i.g.) for 21 days	Upregulate of E-cadherin and downregulation vimentin, attenuate the EMT; reduce the elevated phosphorylation of AKT and GSK3β, prevent the translocation of β-catenin, repress the AKT/GSK3β/β-catenin signaling pathway (Liu et al., 2021)
Isorhamnetin	OVA-induced asthmatic mice	50, 150 mg/kg (i.p.) for 14 days	CysLT1, CysLTR1, IL-4, IL-5, IL-13, TNF-α; NFATc4, ICAM-1 and VCAM-1↓; inhibit calcineurin/NFATc4 pathway (Zhu et al., 2021)
Isorhamnetin	BLM-induced pulmonary fibrosis mice HBECs	10, 30 mg/kg (i.g.) for 28 days 25, 50 and 100 μM for 48 h	EMT, Collagen I, α-SMA, CHOP and eIF2α phosphorylation in pulmonary fibrosis mice↓; inhibit collagen I, α-SMA, GRP78 and CHOP; and reverse the TGFβ1-induced EMT in A549 and HBECs (Zheng et al., 2019)
Kaempferol	OVA-induced allergic airway inflammation guinea pigs	20 mg/kg (i.g.) for 21 days	Notably downgrate the cough reflex response and suppress cough to reach the baseline level in healthy group (Molitorisova et al., 2021)
Kaempferol	OVA-induced asthmatic mice BEAS-2B cells	20 mg/kg (i.g.) for 5 days 1, 5, 10, 20 and 40 μg/mL for 24 h	EOS, lymphocytes and neutrophils↓; the levels of IL-4, IL-5 and IL-13↓; IL-25, IL-33, NOX4, ATG5, Beclin-1↓,suppress airway inflammation through regulating NOX4-mediated autophagy in the OVA-induced mice (Xu et al., 2023)
Kaempferol	OVA-induced asthmatic mice BEAS-2B cells	10, 20 mg/kg (i.g.) for 3 days 1–20 µM for 48 h	Suppress BEAS-2B cells, eosinophil recruitments, attenuate MCP-1 transcription and NF-κB activation, and eotaxin-1 and EMBP production; administrate NF-кB signaling (Gong et al., 2012)
Kaempferol	OVA-induced ARh mice Eol-1 cells	0.2, 2, and 20 mg/kg (i.g) for 10 days 0.2, 2 and 20 μg/mL for 1 h	Reduce the eosinophil and mast cell infiltration; histamine, IgE, IL-32, TSLP, IL-4, IL-8, ICAM-1, MIP-2, and COX-2↓; decreased caspase-1 activation (Oh et al., 2013)
Formononetin	HDM-induced asthmatic mice 16HBE	25 mg/kg (i.g.) for 5 days 5, 10, and 20 μM 2 h	IL-4, Il-6, Il-17A and IgE in BALF↓; enhance the proliferation and migration of LPS-stimulated 16HBE cells lower the Bax/Bcl-2 ratio; inhibite TLR4 and ESR1)/Nod-like receptor; restraine the ESR1/NLRP3/Caspase-1 signaling in LPS-induced 16HBE cells; suppressi ESR1/NLRP3/Caspase-1 signaling (Zhang et al., 2023)
Formononetin	OVA-induced allergic asthmatic mice	10, 20 and 40 mg/kg (i.g.) for 28 days	Regulate Th17/Treg cell balance; the proportion of Th17 and EOS↓; IL-4, IL-5, IL-13, IgE, CCL5, CCL11 and IL-17A↓; ROS↓ and SOD↑; NF-kB, p-NF-κB, JNK and p-JNK↓; upregulating the antioxidative signaling pathway of Nrf2/HO-1 (Yi et al., 2020)

Table 3 summarizes the anti-inflammatory, immunomodulatory, and anti-fibrotic effects of key flavonoid etabolites of AR (e.g., TFA, quercetin, calycosin, isorhamnetin, kaempferol, formononetin) in preclinical models of ARDs, with a focus on their modulation of signaling pathways (e.g., NF-κB, MAPK, calcineurin/NFATc4) and relevant references.

Abbreviations: AR, Astragalus radix; ARDs, allergic respiratory diseases; ARh, Allergic Rhinitis; TFA, total flavonoids of Astragalus; CG, calycosin-7-O-β-D-glucoside; OVA, ovalbumin; i.p., intraperitoneal; i.g., intragastric; LPS, lipopolysaccharide; iNOS, inducible nitric oxide synthase; COX-2, cyclooxygenase-2; MAPK, mitogen-activated protein kinase; NF-κB, nuclear factor-κB; EOS, eosinophils; IL, interleukin; TNF-α, tumor necrosis factor-α; IgE, immunoglobulin E; Th, T helper; Treg, regulatory T cell; EMT, epithelial-mesenchymal transition; AKT, protein kinase B; GSK3β, glycogen synthase kinase 3β; CysLT1, cysteinyl leukotriene 1; NFATc4, nuclear factor of activated T cells c4; BLM, bleomycin; HDM, house dust mite; Nrf2, nuclear factor erythroid 2-related factor 2; HO-1, heme oxygenase 1.

Collectively, APS, astragalosides (AS-II, AS-IV, AS-VII, Mac B), and flavonoids (calycosin, formononetin, quercetin, kaempferol, isorhamnetin)—key bioactive metabolites of AR—consistently inhibit inflammatory responses, AHR and restore immune homeostasis, while ameliorating goblet cell hyperplasia and airway remodeling (e.g., subepithelial fibrosis, airway smooth muscle hypertrophy) caused by chronic inflammation in murine models of allergic asthma. Notably, these anti-remodeling effects improve airway structural integrity, a strong prognostic indicator for asthma progression and long-term outcomes, providing a scientific basis for the clinical application of AR in allergic asthma.

### 4.2 Pharmacodynamics of AR’s metabolites in ARh

ARh is a non-infectious chronic inflammatory nasal mucosal disease primarily mediated by IgE, due to an allergen allergens., often come together with asthma and other allergic diseases (Cheng et al., 2018). Aberrant activation of inflammatory cells and excessive release of proinflammatory mediators are the core mechanisms in ARh, which lead to cellular inflammatory infiltrates, epithelial barrier alterations, glandular secretion and increased vessel permeability. So that ARh often manifests as sneezing, rhinorrhea, and nasal congestion/obstruction (Wise et al., 2023).

#### 4.2.1 Effects of APS

APS has the ability of regulating inflammatory cell activation and cytokine release, as well as ameliorate oxidative stress resulted from ARh. Firstly, Wang and his members founded that in a murine model of ARh, intranasal administration of APS/Chitosan (5, 10, 15 mg/kg) alleviated EOS infiltration in the nasal mucosa, which was associated with reduced expression of IL-4 (a key Th2 cytokine) in nasal mucosal tissues (Wang et al., 2020) (Table 1). Additionally, He revealed in a guinea pig model of ARh, intragastric administration of APS (25, 50, 100 mg/kg) increased the proportion of CD25^+^Foxp3^+^ regulatory T cells (Treg)—a subset that suppresses excessive immune responses—while decreasing the proportion of CD4^+^IL-17^+^ Th17 cells (a pro-inflammatory subset). This dual effect effectively restored the Th17/Treg balance, thereby mitigating immune-mediated nasal inflammation (He et al., 2022) (Table 1). Furthermore, Chen discovered that the homogeneous polysaccharide fraction APS-B1 was shown to downregulate the expression of inducible nitric oxide synthase (iNOS)—an enzyme closely linked to oxidative stress and inflammatory damage—in ARh-related models, further supporting APS’s anti-inflammatory role (Chen et al., 2023) (Table 1).

#### 4.2.2 Effects of AS-IV

Among saponin, AS-IV plays a major role in ARh. It exerts the migration of inflammatory cells and mucus secretion by acting on different cells. Chen found that in OVA-induced ARh mice, intraperitoneal injection of AS-IV (40 mg/kg) significantly reduced the local counts of EOS, mast cells, IL-4 and IL-5 in nasal mucosal tissues and nasal lavage fluid, increased the content of INF-γ, while also lowering reactive oxygen species (ROS) levels in both nasal mucosa and spleen tissues—suggesting AS-IV’s effects on inflammatory cell recruitment and oxidative stress (Chen et al., 2021). Besides, Guo and Xu conducted vitro studies and further demonstrated that AS-IV (20, 40, 60 µM) inhibited the overproduction of mucin five subtype AC (MUC5AC)—a major metabolite of excessive nasal mucus—in histamine (His)-stimulated nasal epithelial cells (NECs), and regulated the expression of inflammation-related genes in NECs and inhibited the secretion of motif chemokine, IL-6, IL-8, MCP-1 and interleukin-1β (IL-1β), thereby targeting mucus hypersecretion (a hallmark of ARh symptoms) (Guo and Xu, 2021) (Table 2). Moreover, AS-IV has also been confirmed to target T cell subset dysregulation (e.g., Th17 overactivation, Treg suppression and Th2 polarization), which is a core pathological feature of ARh: (1) In ARh models, intragastric AS-IV (25, 50 mg/kg) upregulated the expression of Foxp3 (a Treg-specific transcription factor) and TGF-β1, IL-10 (cytokines secreted by Treg cells), while downregulating RORγt (a Th17-specific transcription factor) and IL-17 (cytokines secreted by Th17 cells)—thereby collectively restoring Th17/Treg balance (Zhang and Tian, 2022). (2) AS-IV (25, 50 mg/kg, i.g.) downregulated GATA-3 expression levels, whereas upregulated T-bet expression levels in nasal mucosal of OVA-induced ARh mice model, so as to implicate in the regulation of Th1/Th2 differentiation (Li K. et al., 2017) (Table 2).

#### 4.2.3 Effects of flavonoids

Flavonoids in AR, especially quercetin, calycosin and kaempferol, play anti-inflammatory roles in mitigating ARh. For quercetin, Sagit founded that intraperitoneal injection of quercetin (80 mg/kg) in OVA-induced ARh mice led to a significant decrease in EOS counts, as well as reduced levels of cyclooxygenase-2 (COX-2, a pro-inflammatory enzyme) and serum total immunoglobulin E (IgE)—a key mediator of Type 1 hypersensitivity (Sagit et al., 2017) (Table 3). Ke focused on the relationship between quercetin and T cells. He demonstrateed that quercetin regulates both Th1/Th2 and Th17/Treg balances through modulating key transcription factors. Oral administration of quercetin (20, 35, 50 mg/kg) in ARhs models not only alleviated clinical symptoms (e.g., sneezing, rhinorrhea) but also restored both Th1/Th2 and Th17/Treg balance—validating its role as a broad-spectrum modulator of T cell-mediated immune responses (Ke et al., 2023) (Table 3). For calycosin, Dong discovered that calycosin glycoside (CG, 5, 12.5, 25, 50, 100 µM) exerted anti-inflammatory effects by inhibiting the activation of RAW 264.7 macrophages (a critical cell type in innate immune responses), highlighting its role in suppressing early inflammatory cascades (Dong et al., 2018) (Table 3). For kaempferol, Oh confirmed the anti-inflammatory potential of it through experiments. Oral administration of kaempferol (0.2, 2, 20 mg/kg) in ARh mice reduced caspase-1 activity (a key mediator of pyroptosis, a pro-inflammatory cell death pathway), reduced IL-4, TSLP secretion, increased IFN-γ secretion and attenuated the infiltration of EOS and mast cells in the nasal mucosa (Oh et al., 2013) (Table 3).

### 4.3 Pharmacodynamics of AR’s metabolites in HP

HP is an inflammatory and/or fibrotic disease affecting the lung parenchyma and small airways, typically resulting from ingaling overt or occult antigen in susceptible individuals. Sensitization is of critical importance in the pathogenesis of HP which causes immunological dysregulationa. HP presents as predominantly lymphocytic and granulomatous inflammation in early stage, while augmented alveolar epithelial apoptosis and abnormal fibroblast activity in chronic stage (Raghu et al., 2020). HP is a common cause of progressive pulmonary fibrosis which is closely linked to progressive respiratory dysfunction and reduced survival (Koschel et al., 2025; Fernández Pérez et al., 2021). Given this clinical significance, lots of studies have focused on the anti-fibrotic effects of AR and its bioactive metabolites during the chronic fibrotic phase of HP. Due to the scarcity of established HP models, the above mechanistic evidence was obtained from BLM-induced pulmonary fibrosis or idiopathic pulmonary fibrosis models, which share similar pathological features of chronic inflammation and progressive lung fibrosis with HP. Caution is therefore warranted when extrapolating these findings to HP.

#### 4.3.1 Effect of APS

On one hand, APS exerts anti-fibrotic effects by targeting inflammatory cascades in HP. Wei found that in bleomycin (BLM) pulmonary fibrosis models (a classic model mimicking HP-related fibrosis; caution is warranted when extrapolating these findings to HP), i.g. administration of APS (25, 50, 100 mg/kg) reduced the expression of pro-inflammatory cytokines (e.g., TNF-α, IL-6) and mitigated lung tissue damage. Mechanistically, this was attributed to the inhibition of the toll-like receptor 4 (TLR4)/nuclear factor-κB (NF-κB) pathway—a key mediator of inflammation-driven fibrosis—thereby attenuating pulmonary fibrosis progression (Wei et al., 2023) (Table 1). On the other hand, APS exerts anti-fibrotic effects by targeting fibrotic signaling pathways. Zhang discovered that in a BLM-induced pulmonary fibrosis model (a classic model mimicking HP-related fibrosis; caution is warranted when extrapolating these findings to HP), APS (200 mg/kg, i.g. or 50 mg/mL) exerted multi-faceted anti-fibrotic effects: it mitigated BLM-induced upregulation of collagen and fibronectin (major ECM metabolites), reversed TGF-β1-induced EMT (a critical process driving fibrosis), and suppressed NF-κB pathway activation. These effects were accompanied by reduced expression of mesenchymal markers (vimentin, α-SMA) and increased expression of the epithelial marker E-cadherin—confirming APS’s role in restoring epithelial phenotype and inhibiting mesenchymal transition (Zhang et al., 2020) (Table 1).

#### 4.3.2 Effect of AS-IV

AS-IV exhibits robust anti-fibrotic activity through breaking the “oxidative stress-inflammation-fibrosis” cycle. Doctor Yu conducted an experiment that showed AS-IV (10, 20 and 50 mg/kg, i.p.) downregulated lung tissue levels of malondialdehyde (MDA, a marker of oxidative damage) and reactive oxygen species (ROS), while upregulating antioxidant indices (superoxide dismutase [SOD], total antioxidant capacity [T-AOC]). Concurrently, it reduced the levels of pro-inflammatory cytokines (TNF-α, IL-1β, IL-6) in bronchoalveolar lavage fluid (BALF) in BLM-induced pulmonary fibrosis rats (a classic model mimicking HP-related fibrosis; caution is warranted when extrapolating these findings to HP) (Yu et al., 2016) (Table 2). At the same time, Li found that i.g. administration of AS-IV (10 mg/kg) lowered lung tissue levels of type III collagen, hydroxyproline (a specific marker of collagen synthesis), and α-SMA (a marker of myofibroblast activation), while decreasing serum levels of ECM metabolites (laminin [LN], hyaluronic acid) and high-mobility group box 1 (HMGB1, a pro-fibrotic alarmin). These changes collectively attenuated BLM-induced ECM deposition—a hallmark of advanced fibrosis (note: this study used a BLM-induced pulmonary fibrosis model, not a standardized HP model) (Li L. C. et al., 2017) (Table 2). Furthermore, Qian carried out a study in a BLM-induced pulmonary fibrosis model (a classic model mimicking HP-related fibrosis; caution is warranted when extrapolating these findings to HP), which discovered that i.g. administration of AS-IV (20 mg/kg) alleviated collagen deposition and oxidative stress, suppressed the release of pro-inflammatory cytokines (TNF-α, IL-6), and reversed BLM-induced EMT. These effects were mediated by the inhibition of the TGF-β1/PI3K/AKT pathway—a central signaling axis regulating EMT and ECM synthesis (Qian et al., 2018) (Table 2).

#### 4.3.3 Effects of flavonoids

Previous studies were conducted on flavonoids, especially calycosin and isorhamnetin. Calycosin was identified as a potential anti-fibrotic agent. Liu demonstrated that in preclinical models (BLM-induced pulmonary fibrosis models mimicking HP-related fibrosis; caution is warranted for extrapolation to HP), i.g. administration of calycosin (7, 14 mg/kg) prevented pulmonary fibrosis by inhibiting EMT. This effect was mediated by the suppression of the Akt/glycogen synthase kinase 3β (GSK3β)/β-catenin signaling pathway—an axis that regulates EMT initiation and myofibroblast differentiation (Liu et al., 2021) (Table 3). Zheng discovered that isorhamnetin (10, 30 mg/kg, i.g.) significantly reduced the production of collagen I and α-SMA in BLM-treated pulmonary fibrosis mice (a classic model mimicking HP-related fibrosis; caution is warranted when extrapolating these findings to HP), so as to significantly attenuated matrix protein deposition in the lungs (Zheng et al., 2019).

### 4.4 Pharmacodynamics of AR’s metabolites in AC

AC is a kind of ARDs characterized by chronic irritative cough occurred in atopic individuals. While compared to asthma or ARh, AC has normal sputum EOS, no AHR (Lai et al., 2013; Xu and Ren, 2019). AR and its metabolites have therapeutic effects on AC.

Molitorisova discovered that in a guinea pig model of ovalbumin (OVA)-induced allergic airway inflammation (a surrogate for AC-related airway hypersensitivity). i.g. administration of kaempferol (20 mg/kg once) downgrated the cough reflex response notably elevated in the OVA-induced allergic airway inflammation Besides, long-term administration of kaempferol (20 mg/kg body weight, p.o.) exerted a robust antitussive effect in preclinical models. Specifically, it restored the elevated cough reflex to the baseline level observed in healthy control animals, indicating its potential to normalize cough hypersensitivity—a core feature of AC (Molitorisova et al., 2021) (Table 2). Besides, Li found that Total flavonoids of Astragalus (TFA) exerted anti-inflammatory effects relevant to the management of inflammation-driven chronic cough (including AC). *In vitro* studies showed that TFA downregulated the expression of pro-inflammatory mediators—including TNF-α, IL-1β, IL-6, inducible nitric oxide synthase (iNOS), and cyclooxygenase-2 (COX-2)—in lipopolysaccharide (LPS)-stimulated RAW264.7 macrophages. This inhibition of inflammatory cascades supports TFA’s potential role in alleviating inflammation-driven chronic cough (Li et al., 2018) (Table 3). As the sovereign botanical drug (core metabolite) in the *Astragali Radix Antiasthmatic Decoction* (a classic TCM formula for respiratory disorders), AR has demonstrated efficacy in chronic cough models. Xu vertified that the TCM formula significantly reduced eosinophilic airway inflammation and airway surrounding collagen deposition—pathological changes linked to cough persistence and airway remodeling (Xu et al., 2013b).

Compared with the previous three diseases, there are relatively fewer animal experimental studies on AC. This may be attributed to the difficulty in simulating a pure AC model relying solely on current drugs. However, with changes in the social environment, the number of people with AC is gradually increasing. AR, as a potent therapeutic agent for ARDs, makes it necessary to conduct more relevant studies in the future.

## 5 Effects of AR active metabolites on regulation of signaling pathway in ARDs

In the first half of this article, we summarized the phytochemical profiles and pharmacodynamic effects of AR extracts and their monomeric metabolites. To further elucidate the molecular mechanisms underlying AR’s therapeutic effects on ARDs, the following section focuses on core signaling pathways targeted by AR—including NF-κB, MAPK, TGF-β1/Smad, Nrf2/ARE, NLRP3/Caspase-1, and PI3K/AKT—with a detailed discussion of their regulatory roles.

### 5.1 NF-κB signaling pathway

The NF-κB signaling pathway is a central mediator of immune and inflammatory responses to allergen stimulation. Its aberrant activation drives key pathological processes in ARDs, including excessive inflammation, tissue fibrosis, and immune dysregulation—making it a critical therapeutic target for ARDs such as allergic asthma (Christman et al., 2000), ARh (Ke et al., 2023), and HP (Wei et al., 2023). Below is a detailed overview of how AR bioactive metabolites regulate the NF-κB pathway.

#### 5.1.1 Regulation by APS and its fractions

APS exerts protective effects against airway inflammation and fibrosis primarily by inactivating the NF-κB pathway: Firstly, in OVA-induced allergic rhinitis guinea pigs, APS was shown to inhibit the nuclear translocation of NF-κB p65—a key step in NF-κB activation—thereby suppressing downstream pro-inflammatory cytokine production and alleviating airway inflammation (He et al., 2022). Secondly, in a BLM-induced idiopathic pulmonary fibrosis (IPF) mouse model (mimicking fibrotic progression in chronic HP; caution is warranted when extrapolating these findings to HP), APS reduced collagen deposition and downregulated the expression of toll-like receptor 4 (TLR4), phosphorylated p65 (p-p65)/total p65, and IκBα. These effects confirmed that APS mitigates pulmonary fibrosis by inhibiting the TLR4/NF-κB signaling axis (Wei et al., 2023). Thirdly, at the fraction level, the homogeneous polysaccharides APS-A1 and APS-B1 also target the NF-κB pathway: in LPS-stimulated RAW264.7 macrophages, they significantly reduced the phosphorylation levels of IκBα and p65—blocking NF-κB activation and subsequent inflammatory responses (Chen et al., 2023).

Notably, most studies investigating APS-mediated regulation of TLR4/NF-κB signaling did not perform endotoxin testing (LAL assay), polymyxin B neutralization, or endotoxin removal procedures. Therefore, potential artifacts caused by LPS contamination cannot be fully excluded, and the specific regulatory effects of APS on TLR4/NF-κB signaling should be interpreted with caution.

#### 5.1.2 Regulation by AS-IV

AS-IV modulates the NF-κB pathway through the HMGB1/receptor for advanced glycation end products (RAGE) axis, thereby exerting anti-asthmatic effects: (1) In established asthmatic animal models and airway smooth muscle cell (ASMC) models, AS-IV reduced the expression of HMGB1 and RAGE (upstream activators of NF-κB) while decreasing the phosphorylation levels of p65 and IκBα (key NF-κB pathway metabolites). Besides, AS-IV promotes the pyroptosis of hyperproliferative ASMCs—alleviating airway remodeling in asthma (Zhang H. et al., 2024).

#### 5.1.3 Regulation by AR flavonoids

AR flavonoids (e.g., TFA, CG, quercetin, isorhamnetin, kaempferol, formononetin) exert immunomodulatory and anti-inflammatory effects by targeting distinct nodes of the NF-κB pathway: First, in LPS-stimulated RAW264.7 macrophages, TFA significantly inhibited the phosphorylation of IKKα/β and IκBα, as well as the nuclear translocation of NF-κB p65—blocking the entire NF-κB activation cascade (Li et al., 2018). Second, CG reduced the expression of IκBα and p65 in inflammatory models, thereby suppressing NF-κB-mediated pro-inflammatory signaling (Dong et al., 2018). Third, in ARh models, quercetin downregulated the phosphorylation level of IκBα and the nuclear accumulation of p65—attenuating NF-κB-driven nasal inflammation (Ke et al., 2023). Forth, isorhamnetin decreased the phosphorylation levels of IKK, IκBα, and NF-κB p65, inhibiting pathway activation and subsequent inflammatory responses (Ren et al., 2021). Fifth, kaempferol dose-dependently inhibited the expression of eotaxin-1 and eosinophil (EOS) major basic protein by blocking NF-κB transactivation (Gong et al., 2012). Sixth, the phosphorylation of NF-κB was significantly suppressed after formononetin 40 mg/kg treatmentin OVA-induced allergic asthma mice (Yi et al., 2020).

### 5.2 MAPK signaling pathway

The MAPK signaling pathway serves as a central transduction hub in ARDs, governing three core pathological processes: T-cell immune polarization (e.g., Th2 differentiation), the release of pro-inflammatory mediators (e.g., cytokines, chemokines), and airway epithelial/endothelial barrier damage. The pathway comprises three major functional branches—ERK, JNK and p38 MAPK—each with distinct roles: ERK primarily regulates cell proliferation and survival, while JNK and p38 MAPK are key drivers of inflammatory responses. Notably, targeted inhibitors of ERK, JNK, or p38 have been shown to effectively block the cascade amplification of allergic inflammation, validating the MAPK pathway as a therapeutic target for ARDs (Wang et al., 2016; Ren et al., 2021).

#### 5.2.1 Regulation by APS

APS also exert anti-inflammatory and anti-allergic effects on ARDs by modulating the MAPK pathway. In ovalbumin (OVA)-induced asthmatic rat models, APS significantly downregulated the protein expression levels of ERK1/2 and its phosphorylated form (p-ERK1/2), JNK and its phosphorylated form (p-JNK), as well as p38 MAPK and its phosphorylated form (p-p38 MAPK) in lung tissues. By reducing the expression and phosphorylation activation of these key MAPK pathway metabolites, APS ultimately inhibited the aberrant activation of the MAPK signaling cascade (Wang et al., 2016). Besides, APS-A1 (200 μg/mL) and APS-B1 (200 μg/mL) treatment dramatically suppressed the phosphorylation of JNK and ERK (Chen et al., 2023).

#### 5.2.2 Regulation by AR flavonoids

Flavonoids, a major class of bioactive metabolites in AR, exert anti-inflammatory and anti-allergic effects on ARDs primarily by modulating the MAPK pathway, with distinct metabolites targeting specific branches: First, In LPS-stimulated RAW264.7 macrophages (a model mimicking innate immune activation in ARDs), TFA dose-dependently suppressed the excessive phosphorylation of p38 MAPK and JNK—two pro-inflammatory branches of the MAPK pathway. This inhibition directly reduced the production of downstream pro-inflammatory mediators (e.g., TNF-α, IL-6), thereby alleviating macrophage-driven inflammatory responses (Li et al., 2018). Second, in ARDs-relevant models (e.g., bronchial epithelial cells and macrophages challenged with allergens), isorhamnetin inhibited the phosphorylation levels of ERK, JNK, and p38 MAPK proteins. By targeting all three major branches of the MAPK pathway, it comprehensively suppressed inflammatory signal transduction and subsequent immune hyperactivation (Ren et al., 2021). Third, similar to isorhamnetin, CG reduced the phosphorylation of ERK, JNK, and p38 MAPK in allergen-stimulated bronchial epithelial cells and macrophages. This multi-branch regulation attenuated epithelial barrier damage and inflammatory mediator release, contributing to its anti-ARDs effects (Dong et al., 2018). Forth, in OVA-induced asthmatic mice, formononetin has been shown to dramatically inhibit the activation of the JNK branch (a key mediator of asthmatic airway inflammation). By suppressing JNK-dependent signaling, formononetin reduced airway EOS infiltration and mucus hypersecretion—core pathological features of allergic asthma (Yi et al., 2020).

Collectively, these findings demonstrate that AR-derived APS and flavonoids target the MAPK pathway through branch-specific or multi-branch regulation, thereby disrupting the “signal activation—inflammation—tissue damage” cascade in ARDs.

### 5.3 TGF-β1/Smads signaling pathway

The TGF-β1/Smads signaling pathway exhibi bidirectional regulatory roles in ARDs under physiological conditions, it supports immune tolerance induction and airway tissue barrier repair (a protective function) (Deng et al., 2024); however, its aberrant overactivation drives pathological processes such as airway remodeling, pulmonary fibrosis, and EMT—key contributors to ARDs progression (e.g., chronic asthma, fibrotic HP). Targeting the excessive activation of this pathway has thus emerged as a precise therapeutic strategy for ARDs characterized by fibrosis or remodeling.

#### 5.3.1 Regulation by APS and its derivatives

APS exerts anti-fibrotic and anti-remodeling effects by inhibiting overactive TGF-β1/Smad signaling In OVA-induced asthmatic rats, intraperitoneal injection of APS (150, 200, 300 mg/kg) dose-dependently downregulated the mRNA expression levels of TGF-β1, Smad2, and Smad3—key pro-fibrotic metabolites of the pathway. This suppression reduced TGF-β1-mediated airway smooth muscle hyperplasia and collagen deposition, alleviating asthmatic airway remodeling (Wang et al., 2024). At the same time, in BLM-induced pulmonary fibrosis mice (a classic model mimicking HP-related fibrosis; caution is warranted when extrapolating these findings to HP), APS downregulates the expression of downstream E-cadherin, vimentin, and α-SMA by inhibiting TGF-β1/Smad signaling pathway. These changes reduce collagen deposition and inhibit fibroblast transformation, thereby reversing lung tissue structural disorder, which is the core improvement of fibrotic ARDs pathology (Zhang et al., 2020).

#### 5.3.2 Regulation by AS-IV

AS-IV targets the TGF-β1/Smad pathway to inhibit airway inflammation and AHR in ARDs: In both OVA-induced asthmatic mice and OVA-induced ARh mice, AS-IV downregulated the mRNA expression of TGF-β1, α-SMA, which is correlated with the degree of subepithelial fibrosis, and thymic stromal lymphopoietin (TSLP), which amplifies TGF-β1-mediated immune dysregulation. By suppressing this TGF-β1 axis, AS-IV inhibited the progression of airway inflammation and reduced AHR, two hallmark features of ARDs (Zhang and Tian, 2022; Du et al., 2008).

#### 5.3.3 Regulation by AR flavonoids

AR flavonoids (e.g., isorhamnetin, formononetin, calycosin) primarily target the TGF-β1/Smad pathway to inhibit EMT and pulmonary fibrosis: Firstly, in both *in vivo* (BLM-induced pulmonary fibrosis mice, a classic model mimicking HP-related fibrosis) and *in vitro* (human bronchial epithelial cells [HBECs] treated with TGF-β1) models, isorhamnetin exerted anti-fibrotic effects by: reducing the expression of pro-fibrotic markers (collagen I, α-SMA, vimentin) and restoring the expression of the epithelial marker E-cadherin in BLM-damaged lung tissues; suppressing TGF-β1-induced upregulation of collagen I, α-SMA, glucose-regulated protein 78 (GRP78), and C/EBP homologous protein (CHOP) in HBECs; and reversing TGF-β1-mediated EMT—a critical process that converts epithelial cells to mesenchymal cells, driving fibrosis (Zheng et al., 2019). Secondly, formononetin and calycosin attenuate TGF-β1-driven airway remodeling in a murine asthma model by suppressing EMT in bronchial epithelial cells and limiting the phenotypic transition of epithelial cells toward a pro-fibrotic mesenchymal phenotype, thereby directly mitigating airway remodeling and fibrosis in allergic respiratory diseases (Yang et al., 2016).

In summary, distinct bioactive metabolites of AR (polysaccharides, saponins, flavonoids) target the TGF-β1/Smad pathway through multi-faceted mechanisms—including inhibiting excessive TGF-β1 production, regulating Smad protein phosphorylation/expression, and reversing EMT—thereby normalizing the pathway’s function and ameliorating ARDs pathology (e.g., remodeling, fibrosis, inflammation).

### 5.4 Nrf2/ARE signaling pathway

The Nrf2/ARE signaling pathway is recognized as the core endogenous antioxidant defense pathway. Its protective effects in ARDs are primarily mediated through two synergistic mechanisms: As for antioxidant defense, it promotes the transcription of a battery of antioxidant enzyme genes (e.g., SOD, glutathione peroxidase [GSH-Px]), thereby enhancing cellular antioxidant capacity and inhibiting excessive oxidative stress; As for anti-inflammatory and anti-fibrotic regulation, it alleviates inflammatory injury in lung tissues, reduces inflammatory cell infiltration, and mitigates pulmonary fibrosis caused by alveolar structure collapse (Zheng et al., 2022).

#### 5.4.1 Regulation by APS

APS exerts antioxidant effects on ARDs-relevant cells by activating the Nrf2/ARE pathway. Specifically, in human nasal epithelial cells stimulated with IL-13 (a key Th2 cytokine driving ARh pathology), APS activated the Nrf2/ARE pathway to alleviate IL-13-induced oxidative stress injury—protecting the integrity of the nasal epithelial barrier (Cui et al., 2024).

#### 5.4.2 Regulation by AS-IV

AS-IV targets the Nrf2/ARE pathway to enhance antioxidant capacity and suppress airway inflammation. Studies have demonstrated that AS-IV activates the Nrf2/Keap1 (Kelch-like ECH-associated protein 1) axis—the upstream regulatory module of the Nrf2/ARE pathway. This activation significantly increases the expression levels of Nrf2 and Keap1, thereby improving airway inflammation Relevant evidence was obtained from OVA plus inactivated *Bordetella* pertussis-induced bronchitis in rats (Zhang et al., 2018) and LPS-induced acute lung injury in mice (Zhao et al., 2021), which share similar inflammatory mechanisms with ARDs.

#### 5.4.3 Regulation by flavonoids

Quercetin promotes the nuclear translocation of Nrf2, increasing the nuclear protein expression level of Nrf2 in ARDs-related cells (Zhang M. et al., 2024). Formonetin modulates the Nrf2/ARE pathway through its upstream regulator, sirtuin 1. Specifically, formonetin activates sirtuin 1, which in turn promotes the deacetylation of Nrf2—a post-translational modification that enhances Nrf2 stability and transcriptional activity. Activated Nrf2 then upregulates the expression of heme oxygenase-1 (HO-1), ultimately reducing oxidative stress damage in AR (Huang et al., 2021). At the same time, Isorhamnetin activates the Nrf2/ARE pathway by inhibiting Keap1-dependent Nrf2 degradation. In ARDs models (e.g., allergic asthma), isorhamnetin binds to Keap1, disrupts the Nrf2-Keap1 complex, and promotes Nrf2 nuclear translocation. This activates the transcription of downstream antioxidant enzymes, including SOD and HO-1—enhancing the antioxidant capacity of lung tissues, scavenging excessive ROS, and protecting lung parenchyma from oxidative stress-induced injury (Xu Y. L. et al., 2025).

Collectively, APS, AS-IV and flavonoids activate the Keap1/Nrf2/ARE signaling pathway to trigger the transcription of downstream cell protective genes. This leads to a series of beneficial effects: reduced activity of myeloperoxidase (a marker of oxidative inflammation), decreased MDA content, and significantly increased expression of HO-1 as well as activities of SOD and GSH-Px—collectively restoring redox balance in ARDs.

### 5.5 NLRP3/caspase-1 signaling pathway

The NLRP3/Caspase-1 signaling pathway is a core pro-inflammatory signaling axis in ARDs, with its activation closely linked to the amplification of inflammatory cascades. Recent mechanistic models highlight that targeting NLRP3 inflammasome assembly can modulate inflammatory cell death (pyroptosis), which is closely coupled to NLRP3/Caspase-1 signaling (Kesavardhana and Kanneganti, 2017). Upon activation by allergen or stress signals, the NLRP3 inflammasome oligomerizes and recruits pro-Caspase-1, triggering its cleavage into active Caspase-1. Activated Caspase-1 then mediates the maturation and secretion of pro-inflammatory cytokines (e.g., IL-1β, interleukin-18 [IL-18]), and promotes pyroptosis, a hallmark of inflammatory cell death in ARDs (Huang et al., 2021), ultimately exacerbating airway inflammation and tissue damage in ARDs (Theofani et al., 2022).

#### 5.5.1 Regulation by AS-IV

AS-IV inhibits the NLRP3/Caspase-1 pathway through targeting pyroptosis, oxidative stress and inflammasome activation, as supported by both *in vitro* and *in vivo* studies: AS-IV primarily suppresses the activation step of the NLRP3 inflammasome by inhibiting the expression and assembly of NLRP3 and the cleavage of pro-Caspase-1. On one hand, in LPS-induced inflammatory mouse models (mimicking severe bacterial-induced airway inflammation), AS-IV reversed the LPS-mediated upregulation of Caspase-1 and NLRP3 expression, thereby suppressing the downstream release of IL-1β, IL-18 and ROS, so as to inhibit pyroptosis. Pyroptosis endpoints including Caspase-1 activity and mature IL-1β secretion were examined in this model. On the other hand, AS-IV lowered the serum levels of IL-1β and TNF-α in mice, and ameliorated alveolar collapse and thickening of the lung interstitium (Xu Z. H. et al., 2025). These effects collectively alleviate oxidative stress-driven inflammasome activation and subsequent airway inflammation.

#### 5.5.2 Regulation by formononetin

Formononetin modulates the NLRP3/Caspase-1 pathway by targeting estrogen receptor 1 (ESR1), a regulator of NLRP3 inflammasome activity. In allergic airway epithelial and immune cells, formononetin mainly affects the priming step by downregulating ESR1 and reducing the expression of NLRP3 and pro-IL-1β. In HDM-induced asthmatic mice (a clinically relevant ARDs model), formononetin downregulated the expression of ESR1, NLRP3, and Caspase-1. This multi-target regulation exerted two key therapeutic effects: On one hand, it facilitated the repair of the airway epithelial barrier (a critical defense against allergens). On the other hand, it suppressed the ESR1/NLRP3/Caspase-1 signaling cascade—collectively attenuating airway inflammation and reducing epithelial damage (Zhang et al., 2023).

In summary, targeting the NLRP3/Caspase-1 signaling pathway--via inhibition of inflammasome activation, reduction of oxidative stress, and promotion of epithelial barrier repair—effectively alleviates airway inflammation, mitigates oxidative damage, and reduces airway epithelial/mucosal injury in ARDs. Of note, AR metabolites regulate distinct steps of the NLRP3 inflammasome: AS-IV targets the activation step, while formononetin modulates the priming step. Pyroptosis-related endpoints were assayed in the included models, consistent with current mechanistic understanding of NLRP3-mediated inflammatory cell death.

### 5.6 PI3K/AKT signaling pathway

The PI3K/AKT signaling pathway is a key signal transduction axis regulating cell survival, airway inflammation, metabolism, and tissue remodeling (Gupta et al., 2024). Its aberrant overactivation drives multiple pathological processes in ARDs, such as exacerbating airway inflammation, airway remodeling and pulmonary fibrosis. Targeting the excessive activation of PI3K/AKT and its downstream branches has thus become a critical strategy for alleviating ARDs pathology.

The PI3K/AKT signaling pathway is a key signal transduction axis regulating cell survival, airway inflammation, metabolism, and tissue remodeling. Its aberrant overactivation drives multiple pathological processes in ARDs, such as exacerbating airway inflammation, airway remodeling and pulmonary fibrosis, and this pro-pathological role is further supported by evidence that PI3K/AKT acts as a downstream effector of TGF-β/Smad3 signaling in mediating smooth muscle cell proliferation and fibrotic tissue hyperplasia (Suwanabol et al., 2012). Targeting the excessive activation of PI3K/AKT and its downstream branches has thus become a critical strategy for alleviating ARDs pathology, and the cross-talk between PI3K/AKT and TGF-β/Smad3—an essential regulatory axis in fibrosis (Suwanabol et al., 2012)—further highlights the therapeutic value of targeting this pathway for ARDs with fibrotic remodeling features.

#### 5.6.1 Regulation by APS

APS targets the PI3K/AKT pathway to suppress allergic asthma pathology. Using a combination of network pharmacology and *in vivo* experiments (Hu et al., 2025) confirmed that in OVA-induced asthmatic mice, APS significantly downregulated the phosphorylation levels of PI3K (p-PI3K) and AKT (p-AKT). By inhibiting the activation of the PI3K/AKT pathway, APS alleviated key pathological symptoms of allergic asthma, including EOS infiltration, mucus hypersecretion, and airway remodeling.

#### 5.6.2 Regulation by AS-IV

AS-IV exerts anti-asthmatic and anti-fibrotic effects by targeting multiple nodes of the PI3K/AKT pathway, with context-dependent mechanisms in different ARDs models. One one hand, in OVA-induced asthmatic mice, AS-IV inhibited the phosphorylation of AKT and its downstream mTORC1 substrates—including S6 ribosomal protein and p70 S6 kinase. This suppression of the PI3K/AKT/mTORC1 axis reduced mTORC1 activity, blocked the overproduction of Th2 cytokines, and ultimately alleviated airway inflammation and AHR (Jin et al., 2017). On the other hand, in BLM-induced pulmonary fibrosis models (a classic model mimicking HP-related fibrosis; caution is warranted when extrapolating these findings to HP), AS-IV exerted two complementary anti-fibrotic effects: Li found that AS-IV downregulated miR-21 expression, and by inhibiting the miR-21-mediated PI3K/AKT/mTOR pathway, it activated autophagy and apoptosis to mitigate pulmonary fibrosis (Li et al., 2024); while Qian discovered that AS-IV strongly inhibited the phosphorylation of forkhead box O3a (FOXO3a) at both Thr32 and Ser253 sites, restored FOXO3a activity (a negative regulator of fibrosis), and suppressed AKT phosphorylation in BLM-induced pulmonary fibrosis rats. Concurrently, AS-IV inhibited EMT in alveolar epithelial A549 cells, further preventing fibrotic progression (Qian et al., 2018).

#### 5.6.3 Regulation by calycosin

Calycosin inhibits the AKT/GSK3β/β-catenin branch of the PI3K/AKT pathway to prevent pulmonary fibrosis, and this targeted inhibition interrupts the downstream signaling cascade of TGF-β/Smad3-mediated PI3K/AKT activation, which is a key driver of fibrotic remodeling and EMT (Suwanabol et al., 2012). In BLM-induced pulmonary fibrosis models (a classic model mimicking HP-related fibrosis; caution is warranted when extrapolating these findings to HP), calycosin reduced the BLM-mediated elevation of p-AKT and p-GSK3β. Decreased GSK3β phosphorylation prevented the translocation of β-catenin from the cytoplasm to the nucleus—an event that drives EMT and fibroblast activation. This sequence of effects ultimately repressed the activity of the AKT/GSK3β/β-catenin pathway, mitigating EMT and pulmonary fibrosis (Liu et al., 2021).

### 5.7 Additional relevant signaling pathways

In addition to the core pathways discussed above, JAK/STAT and Calcineurin/NFATc4 also play critical roles in allergic respiratory pathogenesis. Although these two pathways are essential, direct evidence demonstrating their regulation by AR-derived bioactive metabolites in allergic respiratory diseases remains relatively limited compared with major pathways such as NF-κB, NLRP3, PI3K/AKT, and TGF-β/Smad. Therefore, they are summarized in this section as additional relevant signaling axes, whose activity is directly modulated by AR metabolites in the cited ARDs models.

#### 5.7.1 JAK/STAT signaling pathway

The Janus kinase/signal transducer and activator of transcription (JAK/STAT) signaling pathway regulates multiple biological processes critical to the pathogenesis of pulmonary inflammation and fibrosis, including pro-inflammatory response initiation, oxidative stress amplification, and dysregulated cell apoptosis (Almeida-Oliveira et al., 2019; Wang J. et al., 2019). In ARDs, the JAK2/STAT6 branch is of particular importance, as it mediates Th2 cytokine-driven allergic inflammation.

AS-IV targets the JAK2/STAT6 pathway to alleviate allergic inflammation: AS-IV is able to significantly inhibit the upregulation of IL-4, IL-5 and IL-13 in OVA-induced mice, and reduce the protein expressions of p-JAK2 and p-STAT6 as well (Yang and Wang, 2019; Chen et al., 2021). Kaempferol blunts JAK2 signaling and reduces eotaxin-1 expression (Gong et al., 2012). Previous studies indicated Th2-related cytokines IL-4, five (especially IL-13) were upstream stimulators for JAK2/STAT6 pathway activation and could induce phosphorylation of STAT6, which led to excessive mucus secretion (Pernis and Rothman, 2002; Musa et al., 2024). The JAK2/STAT6 pathway plays an important role in AHR and mucus overproduction (Perkins et al., 2011).

#### 5.7.2 Calcineurin/NFATc4 signaling pathway

The Calcineurin/NFATc4 pathway modulates immune responses in ARDs *via* a Ca^2+^-dependent phosphatase-transcription factor cascade, governing the activation of pro-inflammatory genes and immune cell differentiation.

Isorhamnetin, a flavonoid metabolite of AR, inhibits this pathway to exert anti-ARDs effects: In asthmatic mice models, isorhamnetin reduced the expression of multiple pro-inflammatory mediators, including: Firstly, Th2 cytokines (IL-4, IL-5, IL-13) and pro-inflammatory cytokine TNF-α; Secondly, cysteinyl leukotriene 1 (CysLT1) and its receptor (CysLTR1, a key mediator of airway constriction); Finally, adhesion molecules (ICAM-1, VCAM-1, which promote inflammatory cell recruitment). These effects were attributed to isorhamnetin’s inhibition of the Calcineurin/NFATc4 pathway, which suppressed the transcription of downstream pro-inflammatory genes (Zhu et al., 2021).

**FIGURE 3:**
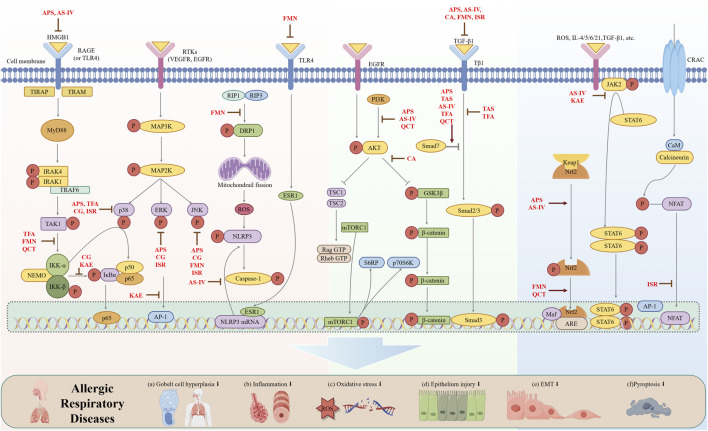
Signaling pathways targeted by major AR metabolites in the treatment of ARDs. This figure summarizes the key signaling pathways modulated by major AR metabolites (APS, AS-IV, quercetin, *etc.*) in ARDs, including NF-κB, MAPK, TGF-β1/Smads, Nrf2/ARE, NLRP3/Caspase-1, PI3K/AKT, JAK/STAT, and Calcineurin/NFATc4 pathways. These metabolites exert therapeutic effects by inhibiting or activating specific targets in these pathways, thereby reducing goblet cell hyperplasia, epithelial injury, inflammation, oxidative stress, EMT, and pyroptosis (supported by representative studies: (Xu et al., 2025a; Wang et al., 2024; Ke et al., 2023; Zhang et al., 2023; Chen et al., 2021; Ren et al., 2021; Zhu et al., 2021; Jin et al., 2017). Abbreviations: AR, Astragalus radix; ARDs, allergic respiratory diseases; APS, Astragalus polysaccharides; AS-IV, astragaloside IV; FMN, formononetin; QCT, quercetin; CA, calycosin; KAE, kaempferol; ISR, isorhamnetin; EMT, epithelial-mesenchymal transition.

In summary, numerous signaling pathways have been demonstrated to orchestrate allergic inflammation, AHR, mucus overproduction, and pulmonary remodeling in ARDs. Bioactive monomers of AR (e.g., APS, AS-IV, isorhamnetin) exert therapeutic effects in allergic respiratory contexts by targeting these pathways—specifically, inhibiting inflammation, oxidative stress, and excessive cell apoptosis, while reducing mucus hypersecretion, collagen deposition, and tissue fibrosis. Key pathways targeted include NF-κB, MAPK, TGF-β1/Smad, Nrf2/ARE, NLRP3/Caspase-1, PI3K/AKT, JAK/STAT, and Calcineurin/NFATc4, all of which are directly linked to allergic respiratory phenotypes in the cited primary literature. The signaling pathways of major metabolites in AR against ARDs are summarized in Figure 3.

## 6 Effects of AR active metabolites on immune homeostasis regulation pathways in ARDs

**FIGURE 4:**
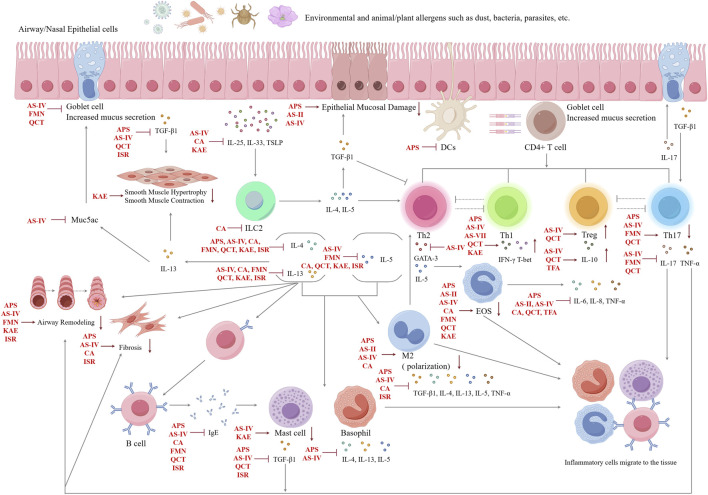
AR active metabolites on immune homeostasis regulation pathways in ARDs. This figure illustrates the modulation of immune homeostasis by AR active metabolites in ARDs, showing the promotion/inhibition of cytokine-immune cell signaling (grey arrows/symbols) and the effects of AR metabolites on immune cell/cytokine levels (maroon arrows/symbols). These regulatory effects restore immune balance and alleviate allergic inflammation (supported by representative studies: (Cui et al., 2024; Zhang and Tian, 2022; Chen et al., 2021)). Abbreviations: AR, Astragalus radix; ARDs, allergic respiratory diseases; FMN, formononetin; QCT, quercetin; CA, calycosin; KAE, kaempferol; ISR, isorhamnetin; M2, M2 macrophage; EOS, eosinophil; DCs, dendritic cells; Treg, regulatory T cell; Th, T helper cell; IgE, immunoglobulin E; IL, interleukin; IFN-γ, interferon-γ; TNF-α, tumor necrosis factor-α; TGF-β1, transforming growth factor-β1; TSLP, thymic stromal lymphopoietin.

Immune homeostasis imbalance, characterized by Th2-type immune dominance (excessive secretion of IL-4/IL-5/IL-13), Th17 cell activation (IL-17-mediated inflammation), and aberrant activation of ILC2, is a hallmark pathological feature of ARDs (Chinese Thoracic Society Chinese Medical Association et al., 2025). Active metabolites of AR with anti-inflammatory and immunomodulatory properties, target these dysregulated immune processes to restore homeostasis. Their regulatory effects are primarily mediated through three core pathways, as detailed below (Figure 4).

### 6.1 ILC2 function regulation pathway

ILC2s are key innate immune cells that drive type 2 inflammation in ARDs, independent of T/B cells. Activated by IL-33, IL-25, or TSLP (type 2 alarmins), ILC2s secrete IL-5 and IL-13—mirroring Th2 cell functions—and amplify allergic inflammation *via* the IL-33/ST2-ILC2-IL-13 axis (Ro et al., 2018; Tian et al., 2024; Rodriguez-Rodriguez et al., 2021). AR metabolites APS and AS-IV target this axis to inhibit ILC2 activation and cytokine secretion, thereby blocking type 2 immune amplification.

In asthmatic mice, APS-induced Nrf2 activation downregulates the expression of ST2 (the cognate receptor for IL-33) on the surface of ILC2. Reduced ST2 expression impairs ILC2 responsiveness to IL-33 (a major activator of ILC2), thereby inhibiting ILC2 activation and the subsequent secretion of IL-13. These effects ultimately alleviate AHR and excessive mucus secretion—two hallmark pathological features driven by ILC2-derived IL-13 (Cui et al., 2024) (detailed in Section 3.4).

AS-IV suppresses the PI3K/AKT/mTOR signaling pathway (detailed in Section 3.6), which is critical for regulating ILC2 glycolytic metabolism. Reduced PI3K/AKT/mTOR activity decreases the glycolytic ener gy supply required for ILC2 proliferation, thereby suppressing ILC2 expansion and the secretion of IL-5 and IL-13. In ARh models, this mechanism also leads to a significant reduction in the number of ILC2 in the nasal mucosa, further mitigating type 2 inflammation (Chen et al., 2021).

### 6.2 Th1/Th2 balance regulation pathway

The Th1/Th2 balance is a cornerstone of adaptive immune homeostasis. In ARDs, this balance is skewed toward Th2 dominance, where Th2 cells secrete IL-4 (drives B cell class switching to IgE), IL-5 (recruits and activates eosinophils), and IL-13 (promotes mucus hypersecretion and AHR). Core metabolites—including AS-IV, APS, and quercetin—modulate Th cell polarization by inhibiting Th2 differentiation, promoting Th1 polarization, and reducing the secretion of Th2-associated cytokines (IL-4/IL-5/IL-13), thereby re-establishing Th1/Th2 homeostasis.

In ovalbumin (OVA)-induced allergic mice, intragastric administration of AS-IV (25–50 mg/kg) corrects Th1/Th2 imbalance through dual regulation of lineage-specific transcription factors: it upregulates T-bet (a master transcription factor driving Th1 differentiation) and downregulates GATA-3 (a Th2-specific transcription factor that promotes IL-4/IL-13 expression). Concurrently, AS-IV enhances immune tolerance by promoting the expression of FOXP3 (a signature transcription factor of regulatory T cells [Treg]), which further suppresses excessive immune responses. These effects collectively reduce eosinophil (EOS) infiltration in the airway and alleviate AHR (Li K. et al., 2017).

In a murine model of ARh, APS directly targets Th2-mediated inflammation by downregulating the expression of IL-4 (a core Th2 cytokine) in the nasal mucosa. This inhibition of IL-4 not only suppresses EOS chemotaxis and activation but also reduces EOS-mediated tissue damage. *In vitro* studies further confirm that APS can directly inhibit IL-13 secretion by primary Th2 cells, thereby blocking IL-13-induced excessive mucus secretion (a key pathological feature of ARDs) (Wang et al., 2020; Cui et al., 2024).

In ARh models, quercetin attenuates Th2-driven allergic responses by inhibiting OVA-induced increase of IgE, histamine, IL-4, and IL-5, and decrease of IFN-γ, which in turn reduces mast cell degranulation and the release of histamine, leukotrienes, and other pro-inflammatory mediators. This sequence of effects further weakens Th2-mediated immune amplification, contributing to the alleviation of allergic symptoms (Ke et al., 2023).

Kaempferol administrated orally significantly downregulated the levels of IL-4 and upregulated IFN-γ levels in ARh mice, regulating the Th1/Th2 balance (Oh et al., 2013).

### 6.3 Th17/IL-17 inhibition pathway

Th17 cells, a subset of CD4^+^ T cells that secrete IL-17A and IL-17F, contribute to severe ARDs by mediating airway inflammation and remodeling (Quan et al., 2023). AS-IV and Kaempferol—two key active metabolites of AR—suppress Th17 cell differentiation and IL-17 secretion, thereby mitigating neutrophil infiltration and neutrophilic airway inflammation.

In OVA-induced asthmatic mice, AS-IV specifically inhibits Th17 polarization by downregulating RORγt (a master transcription factor governing Th17 cell differentiation). Reduced RORγt expression leads to decreased production of IL-17A (the primary effector cytokine of Th17 cells) in lung tissues. Additionally, AS-IV inhibits the expression of neutrophil chemokines (CXCL1 and CXCL2), which are critical for recruiting neutrophils to the airway. By targeting both Th17 cell function and neutrophil chemotaxis, AS-IV effectively relieves neutrophilic airway inflammation (Zhang and Tian, 2022).

In ARh models, oral administration of kaempferol exerts indirect inhibitory effects on Th17 activation by targeting pyroptosis-related pathways. It inhibits the activity of caspase-1 (a key enzyme in pyroptosis), which reduces the cleavage and release of IL-1β (a critical cofactor for Th17 cell differentiation). Reduced IL-1β availability suppresses Th17 cell polarization and IL-17 secretion. Concurrently, kaempferol decreases the infiltration of EOS and mast cells in the nasal mucosa, further synergizing to alleviate allergic airway inflammation (Oh et al., 2013).

In the current study, quercetin reversed the effects of OVA by reducing the levels of IL-17, TGF-β, IL-6, and TNF-α, but elevating the levels of IL-10 and Foxp3, so as to promote the balance of Treg/Th17 cells (Ke et al., 2023).

### 6.4 Synergistic mechanisms among AR active metabolites

Collectively, AR exerts its comprehensive regulatory effects on ARDs immune homeostasis through the complementary and potential synergistic interaction of its core active metabolites (APS, saponins including AS-IV, and flavonoids including quercetin and kaempferol), with each metabolite targeting distinct links of the three aforementioned immune regulation pathways to enhance the overall therapeutic effect. As a foundational metabolite for potential synergy, APS not only directly modulates ILC2 function and Th1/Th2 balance to suppress type 2 inflammation but also enhances the absorption and bioavailability of saponins and flavonoids (as discussed in Section 6.7), thereby laying a solid foundation for their targeted therapeutic effects. AS-IV, as a key saponin metabolite, focuses on inhibiting ILC2 proliferation and Th17 polarization, targeting the amplification of allergic inflammation and airway remodeling. Flavonoids (quercetin, kaempferol) complement these effects by enhancing anti-inflammatory responses, regulating Treg/Th17 balance, and suppressing mast cell degranulation, further reinforcing the restoration of immune homeostasis. Although current evidence for direct synergistic effects (from combined administration in the same experiment) is limited, the multi-metabolite network of AR enables it to simultaneously target multiple links of ARDs immune dysregulation—from ILC2-driven innate inflammation to Th2/Th17-mediated adaptive inflammation. This complementary action achieves a more comprehensive and effective regulation of immune homeostasis than the effects of any single active metabolite alone, which underlies the clinical efficacy of AR in the treatment of ARDs.

## 7 The safety of AR and its active metabolites based ARDs therapy

To comprehensively evaluate the safety of bioactive metabolites in AR, this section summarizes toxicological studies focusing on acute toxicity, chronic toxicity, and specific adverse effects.

### 7.1 Acute toxicity of AR extracts

Acute toxicity studies showed that a single administration of AR extract at doses up to 5,000 mg/kg did not induce lethal effects in experimental animals, indicating a high acute safety margin (Wang P. et al., 2023).

### 7.2 AR total glycoside oral liquid

In both acute and subchronic toxicity studies in mice, the maximum tolerated dose of AR total glycoside oral liquid was 200-fold higher than the typical human equivalent dose (80 mL/kg). All physiological parameters (e.g., hematology, biochemistry, organ weight) remained within normal ranges during the extended testing period, supporting its favorable clinical safety profile (Rios and Waterman, 1997).

### 7.3 Safety of 70% ethanol-extracted AR

Further studies on AR extracts isolated using 70% ethanol (a common extraction solvent for TCM) showed that oral administration of 4,000 mg/kg/day to both male and female rats did not induce any no-observed-adverse-effect level (NOAEL)-related changes (e.g., organ histopathological lesions, abnormal behavior). Additionally, the oral approximate lethal dose of this extract was confirmed to be >5,000 mg/kg, further supporting its high acute safety (Song et al., 2017).

### 7.4 Species-specific dose ranges

For AR extracts, the safe dose range was determined to be 5.7–39.9 g/kg in rats and 2.85–19.95 g/kg in beagles—equivalent to 70-fold and 35-fold the human dose (0.57 g/kg for a 70 kg adult), respectively—providing species-specific safety references for clinical translation (Yu et al., 2007).

### 7.5 The safety profile of the metabolites in AR-based ARDs therapy

#### 7.5.1 APS safety

The maximum tolerance for APS in mice was beyond 15 g/kg (equivalent to 300 times the recommended dose for humans). In the 30-day feeding experiment of rats, after giving APS at doses of 2.5, 5.0, and 7.5 g/kg, the rats grew and developed well. Besides, there were no obvious abnormalities in blood and biochemical indicators, liver, kidney, stomach, and intestinal pathology (Zhou et al., 2018). Liu (Liu et al., 1996) conducted acute toxicity experiments on mice with the maximum injectable dose (6.22 g/kg) of astragalus polysaccharide injection, and the results showed that except for a few transient loose stools, dorsal hair retrograde dampness, and significant increase in spleen, there were no obvious abnormalities in the general condition and organ anatomy of the mice.

#### 7.5.2 Astragalosides safety

AS-II exhibited toxicity in mice at a dose of 500 μg, manifested as 5%–10% body weight loss—indicating potential dose-dependent adverse effects that require attention (Hong et al., 2011). AS-IV safety: Maternal and developmental toxicity: At the same time, study explored the found that maternal and developmental toxicity of AS-IV. During the critical organogenesis period, intravenous administration of AS-IV (0.5–1.0 mg/kg) to Sprague-Dawley rats and New Zealand White rabbits induced dose-dependent maternal toxicity (e.g., reduced food intake, weight gain retardation), though no teratogenic effects (e.g., fetal malformations) were observed (Zhu et al., 2009). Maternal exposure to AS-IV (1.0 mg/kg, 4 weeks) delayed neonatal development in mice, including delayed vibrissae (whisker) development, eye opening, and acquisition of the cliff avoidance reflex. Notably, this exposure did not affect neonatal memory or learning abilities (Wan et al., 2010). Current evidence warrants cautious administration of AS-IV during pregnancy, particularly in the first trimester (critical organogenesis stage).

#### 7.5.3 AR flavonoids safety

Researchers had already explored the safety of TFA, quercetin, calycosin and formononetin. In murine acute oral toxicity studies, the maximum tolerated dose of TFA exceeded 15 g/kg. Further evaluations confirmed no detectable cytotoxicity in somatic cell lines or genotoxicity in germ cells, supporting its safety for short-term use (Wang et al., 2016). Single intravenous (i.v.) doses of quercetin (100–150 mg/kg) or two consecutive doses up to 136 mg/kg did not induce toxicity in rabbits (Ambrose et al., 1952). Besides, in human studies, oral administration of quercetin at doses up to 1,000 mg/day for 12 weeks did not cause significant adverse effects (Harwood et al., 2007). Quercetin and isorhamnetin exhibited low toxicity toward normal mammalian cells, with no obvious cytotoxicity observed in non-tumor cell lines (Naksuriya et al., 2022). What’s more, at low doses (6.25–12.5 μM), calycosin and CG were shown to promote the proliferation of breast cancer cells *in vitro*. However, no adverse effects of these two flavonoids have been reported in normal physiological contexts or clinical settings (Li S. et al., 2017; 2022). Also, i.p. administration of formononetin at 300 mg/kg over 14 days induced acute mortality in mice. The intraperitoneal lethal dose 50% was determined to be 103.6 mg/kg, with a NOAEL of 50 mg/kg body weight. All other acute or sub-acute intraperitoneal doses below the NOAEL were deemed safe (Pingale and Gupta, 2023).

### 7.6 Botanical drug-drug interactions and special population contraindications

AR and its bioactive metabolites show a favorable safety profile in preclinical and clinical practice, yet potential botanical drug-drug interaction risks and administration precautions for special populations require strict attention to ensure rational clinical use. Metabolic studies have demonstrated that AR may interfere with the metabolism of conventional chemical drugs by regulating hepatic drug-metabolizing enzymes and transporters, thus altering the systemic exposure and pharmacodynamic effects of co-administered drugs and inducing botanical drug-drug interaction risks (Wang et al., 2025). A meta-analysis of 31 RCTs involving 2,648 participants confirmed that APS supplementation improves treatment outcomes, enhances immune function, and exhibits high safety in malignant tumor patients, supporting its promising clinical application (Li Q. et al., 2025). However, systemic safety data for AR and its polysaccharides remain insufficient in vulnerable groups (pregnant/lactating women, pediatric patients, and those with severe hepatic/renal dysfunction), with long-term or high-dose administration posing unquantified adverse reaction risks due to the lack of targeted clinical evidence (Li Q. et al., 2025). Current AR safety research has notable limitations: studies on AR-mediated botanical drug-drug interactions only involve preliminary exploration of metabolic enzyme regulation, lacking in-depth clinical evidence on interaction types, risk levels and dose-effect relationships with different drug classes (Wang et al., 2025); safety evaluations (including the above RCT-based meta-analysis) are mostly focused on general and malignant tumor populations, with scarce targeted trials and long-term follow-up data in special groups, which hinders the formulation of standardized administration guidelines (Li Q. et al., 2025). Furthermore, the safety differences of AR with different extraction processes and dosage forms in special populations remain unelucidated, further limiting its precise and safe clinical application.

### 7.7 Pharmacokinetics, oral bioavailability, and human equivalent dose translation of AR metabolites

The pharmacokinetic properties and oral bio-availability of key active metabolites from AR—including APS, AS-IV, and flavonoids--are critical for clinical translation in ARDs. APS shows low oral bio-availability due to high molecular weight and poor membrane permeability, acting mainly in the gastrointestinal tract with limited systemic absorption (Song et al., 2024). AS-IV exhibits extremely low aqueous solubility (only 0.05 mg/mL) and poor intestinal permeability, resulting in an oral bioavailability of merely 7.4% (Zhang et al., 2007; Huang et al., 2006). Flavonoids such as calycosin and isorhamnetin show poor absorption and rapid phase II metabolism. For example, the bioavailability of calycosin-7-O-β-D-glucoside is only 0.304% after oral administration (Li T. Y. et al., 2025; Gong et al., 2021).

Multiple strategies have been verified in animal studies to enhance absorption. Constructing single or composite delivery systems such as lipid carriers, hydrogels, polymer micelles, nanoparticles, and electrospun nanofibers, can improve the stability, safety, and bioavailability of AS-IV formulations (Luo et al., 2023). Ultra-small-size AS-IV-loaded lipid nanocapsules enabled drug release of nearly 80% within 48 h in mice (Sun et al., 2020). The efflux transporter inhibitor Tariquidar increased cellular uptake by 3.3-fold by inhibiting transmembrane efflux of AS-IV (Yao et al., 2026). Quercetin hydrogel scaffold, quercetin liposomes and enzymatically modified quercetin increased its oral bioavailability (Liu et al., 2025). Li created an isorhamnetin-loaded Soluplus-TPGS mixed polymer micelle, which enhanced the oral bioavailability of mixed micelles nearly 3-fold compared with free isorhamnetin (Li Q. et al., 2025). Additionally, Astragalus homogeneous polysaccharide can form complexes with AS-IV and flavonoids so as to enhance their intestinal mucus permeability and absorption, supporting APS as a potential excipient to improve AR bioavailability (Wu et al., 2025; Yue et al., 2022). APS can also self-assemble into porous, high-surface-area aggregates that encapsulate calycosin and formononetin *via* hydrogen bonding, improving their stability, solubility, and intestinal absorption *in vivo* (Yang et al., 2023). These formulation strategies and APS-mediated absorption enhancement can effectively improve the oral bioavailability and pharmacokinetic/pharmacodynamic profiles of AR metabolites, particularly AS-IV, thereby helping to reconcile effective preclinical doses with expected human exposures.

Appropriate conversion from animal doses to human equivalent doses is essential for rational clinical design (FDA, 2005). Advanced delivery systems hold great promise to optimize pharmacokinetic profiles and facilitate clinical translation.

### 7.8 Recommended dose ranges of the active metabolites in AR

Current evidence indicates that not all AR metabolites exhibit dose independence or lack toxic side effects. While metabolites like APS, TFA, and quercetin show favorable safety profiles in recommended dose ranges, others (e.g., AS-IV, formononetin, kaempferol) have potential adverse effects (e.g., maternal toxicity, iron metabolism interference) that require attention. A retrospective analysis of 2,297 TCM prescriptions containing AR (from the Han Dynasty to modern times) identified a wide dosage range of AR from 0.37 g (minimum) to 93.25 g (maximum); interquartile range calculation defined the typical historical dosage as 1.24–42.50 g, which provides a critical reference for the rational design of modern clinical dosages of AR (Cao et al., 2025).

In future studies, researchers must: (1) determine the optimal therapeutic concentration range for each bioactive metabolite to balance efficacy and safety (2) conduct long-term chronic toxicity studies (e.g., 6–12 months); to evaluate potential cumulative effects; and (3) explore metabolite-specific contraindications (e.g., AS-IV in pregnancy, kaempferol in iron-deficient patients) to guide clinical rational use.

Despite the favorable safety profile of AR and its metabolites observed in preclinical studies, several critical aspects remain to be emphasized for clinical translation. First, botanical drug-drug interactions between AR and conventional medications used in ARDs (e.g., inhaled corticosteroids, bronchodilators, biologics) have not been systematically characterized. Potential pharmacokinetic or pharmacodynamic interactions may affect efficacy or safety in combined therapy and warrant further clinical evaluation. Second, most current safety data are derived from animal models; evidence from long-term human studies, clinical adverse event monitoring, and special populations (e.g., children, elderly, pregnant women) remains limited. Third, standardization and quality control of AR products are essential to ensure consistency in efficacy and safety. Variations in planting region, harvesting time, processing methods, extraction procedures, and metabolite purity may lead to differences in pharmacological activity and toxicological risk. Future clinical application of AR in ARDs should consider botanical drug-drug interactions, strengthen post-marketing surveillance in humans, and implement standardized quality control to promote safe and rational use.

## 8 Clinical studies on AR

Among TCM, AR exhibits diverse pharmacological activities and substantial clinical value. Although extensive preclinical research has clarified the mechanisms of AR in treating ARDs and provided abundant experimental evidence, clinical applications of AR-based preparations remain relatively limited. This section summarizes clinical studies involving AR monotherapeutic preparations and AR-containing TCM metabolites, to provide a basis for the further development and translation of AR-derived metabolites.

Clinical studies on AR monotherapeutics have focused on oral and injectable formulations, with dosages and efficacy varying by preparation type (Table summarized from cited studies).

### 8.1 Oral preparations

The clinical dosages of Astragalus Granules (National Medical Product Administration [NMPA] approval nos.: Z20003380, Z20113052) range from 8 to 30 g/day by oral administration (Wang W. et al., 2019; Zhang et al., 2016). A randomized controlled trial (RCT) enrolling 150 children with cough variant asthma demonstrated that oral Astragalus Granules (30 g/day) significantly reduced serum levels of pro-inflammatory and allergic markers (TNF-α, IL-4, EOS, total IgE) and elevated anti-inflammatory IL-10 levels, with the primary endpoint of modulating abnormal inflammatory and allergic responses in pediatric cough variant asthma (Zhang et al., 2016). A clinical study of pediatric asthma in remission stage showed that oral Astragalus Granules (8 g/day) combined with inhaled budesonide (corticosteroid) and terbutaline (β_2_-agonist) improved therapeutic efficacy in asthmatic children; the key endpoint was restoring the Treg/Th17 balance, a core mechanism for preventing asthma exacerbation (Wang W. et al., 2019).

Several other oral AR formulations have also been investigated. An RCT including 80 children with allergic asthma (40 in intervention/40 in control) found that 6-month oral administration of Astragalus Oral Solution significantly reduced serum TGF-β, Th1 cytokines (IL-2, IFN-γ) and Th2 cytokines (IL-4, IL-6), while increasing the proportion of CD4^+^CD25^high^CD127^low^ Treg cells; the primary outcomes were enhanced immune regulation and suppressed allergic airway inflammation (Wang et al., 2018). Besides, A RCT evaluating Astragalus membranaceus for seasonal ARh showed that oral AR capsules (320 mg/day) alleviated core clinical symptoms of ARh and improved patient quality of life, with the primary endpoints of symptom relief and quality of life improvement in ARh patients (Matkovic et al., 2010).

Numerous TCM formulas with AR as a major metabolite have shown high clinical efficacy in the treatment of ARDs, with core therapeutic endpoints of relieving allergic inflammation and airway symptoms; representative formulas include Yu-Ping-Feng Powder (Lin et al., 2025; Chen et al., 2014), Bu-Zhong-Yi-Qi-Tang (Yang and Yu, 2008), Bu-Fei Decoction (Liu and Yang, 2022), Wenfei Zhiliu Dan (Feng et al., 2021), Huangqi Xixin Decoction (Wang et al., 2022) and Jinbei Oral Liquid (AR as sovereign botanical drug, Zhang A. et al., 2025).

### 8.2 Injectable preparations

The recommended clinical dosage of APS Injection (NMPA approval no.: Z20040086): is 250 mg/day by intravenous infusion. An RCT of 80 patients with stable airway inflammation and hyperreactivity (40 in intervention/40 in control) indicated that APS Injection combined with doxofylline effectively relieved cough, enhanced exercise tolerance, and improved key lung function parameters (FEV_1_) and blood gas indices (partial pressure of oxygen); the primary endpoints were lung function and clinical symptom improvement in these patients (Chen and Li, 2024). The product label for Astragalus Injection (NMPA approval no.: Z13020999) specifies a daily dosage of 10–20 mL intravenous infusion or 2–4 mL intramuscular injection, although specific efficacy data in ARDs were not identified in the retrieved literature. The World Federation of Chinese Medicine Societies suggests that AR injection be used for patients with seasonal, perennial, and persistent ARh attacks, especially those who are prone to colds and are afraid of wind and cold (World Federation of Chinese Medicine Societies, 2024).

### 8.3 Combined therapy with AR preparations

An RCT of 90 children with asthma (30 in AR monotherapy/30 in glucocorticoid monotherapy/30 in combination therapy) revealed that AR preparations combined with glucocorticoids achieved a significantly higher total effective rate than either monotherapy; the key outcomes were increased peak expiratory flow rate (PEFR) and Th1 cytokine (IFN-γ) levels, and decreased Th2 cytokine (IL-4) levels, confirming synergistic modulation of the Th1/Th2 balance (Lin et al., 2011).

Overall, available clinical data, including several randomized controlled trials, support the potential value of AR monotherapies and AR-containing TCM metabolites in allergic asthma and allergic rhinitis. Reported outcomes include improvements in clinical symptoms, lung function, inflammatory profiles, immune balance, and quality of life. However, most studies are relatively small-scale, and high-quality large-scale RCTs with long-term follow-up remain limited. Standardized assessments of effect sizes and comprehensive safety profiles are also insufficient. Therefore, current clinical evidence remains encouraging but not yet definitive, and further well-designed clinical investigations are warranted.

## 9 Conclusions and prospects

The global incidence of (ARDs continues to rise, imposing significant socioeconomic and public health burdens worldwide—particularly in China and other low- and middle-income countries (Wang M. et al., 2023; Zhang et al., 2022). AR, a classic botanical drug in TCM, has long been clinically utilized for managing ARDs, with extensive preclinical research validating its therapeutic potential. While existing reviews have touched on AR’s immunomodulatory effects in allergic diseases (Bival Štefan, 2024), few have specifically focused on its mechanisms in ARDs as a unified disease spectrum. This narrative review addresses this gap by systematically synthesizing the immunomodulatory and anti-inflammatory mechanisms of AR and its bioactive metabolites in ARDs, clarifying their targeted signaling pathways and providing a comprehensive foundation for clinical translation.

### 9.1 Key conclusions

AR’s therapeutic effects on ARDs (allergic asthma, ARh, AC, HP) are primarily mediated by three major classes of bioactive metabolites: polysaccharides (e.g., APS), saponins (e.g., AS-II, AS-IV, AS-VII), and flavonoids (e.g., quercetin, calycosin, isorhamnetin, kaempferol, formononetin). Among these, AS-IV and APS have been the most extensively investigated, with consistent evidence demonstrating their robust anti-inflammatory, immunomodulatory, anti-AHR, anti-remodeling, and anti-fibrotic effects.

Mechanistically, these metabolites target multiple core signaling pathways implicated in ARDs pathogenesis, including NF-κB, MAPK, TGF-β1/Smad, Nrf2/ARE, NLRP3/Caspase-1, PI3K/AKT, JAK/STAT, and Calcineurin/NFATc4. Additionally, they restore immune homeostasis by regulating key immune pathways: ILC2 activation, rebalancing Th1/Th2 polarization, and suppressing Th17/IL-17-mediated inflammation. Collectively, these actions alleviate airway inflammation, reduce mucus hypersecretion, mitigate oxidative damage, and halt progressive airway remodeling and pulmonary fibrosis—core pathological processes driving ARDs progression and poor prognosis.

Clinical evidence, including RCTs, supports the efficacy of AR-based preparations (monotherapies and TCM formulations) in improving ARDs symptoms, lung function, inflammatory profiles, and immune balance. However, most clinical studies are small-scale, and high-quality, large-scale RCTs with long-term follow-up remain limited, highlighting the need for further validation of AR’s translational value.

### 9.2 Current limitations

Despite significant advances, research on AR in ARDs faces several critical limitations that require systematic addressing:

#### 9.2.1 Understudied metabolites and signaling pathways

While AS-IV has been extensively investigated, other saponin metabolites (e.g., AS-II, AS-VII) and flavonoid/polysaccharide derivatives (e.g., isorhamnetin, kaempferol, formononetin) have received limited attention in the context of ARDs. Additionally, the regulatory effects of AR on the JAK2/STAT3 pathway—a key mediator of asthma and airway remodeling (Qu et al., 2022)--remain underelucidated, with many targets and mechanisms of understudied metabolites lacking validation through well-designed *in vitro* and *in vivo* experiments.

#### 9.2.2 Insufficient clinical evidence

Most existing clinical studies focus on TCM formulations containing AR, while single-metabolite preparations (e.g., pure APS, AS-IV) have been primarily investigated in oncology, cardiovascular, and cerebrovascular diseases rather than ARDs (Li et al., 2022). Preclinical mechanistic evidence far outweighs clinical data, which is largely confined to small-scale studies of complex TCM formulations. The existing clinical researches—including inconsistent study designs, insufficient sample sizes in preclinical and clinical studies, and lack of long-term follow-up data. Rigorously designed, large-scale human trials with standardized outcome measures (e.g., lung function parameters, inflammatory cytokine levels, quality-of-life scales) are urgently needed to strengthen translational relevance.

#### 9.2.3 Content imbalance across ARDs subtypes

Research is disproportionately focused on allergic asthma and ARh, while high-quality preclinical and clinical evidence for AC and HP remains extremely limited. For HP specifically, although APS (He et al., 2022) and AS-IV (Jin et al., 2017) have been shown to downregulate Th17 cell levels and restore Th17/Treg balance in asthma and ARh, no direct evidence confirms similar effects in HP. This gap is largely attributed to challenges in establishing stable acute HP models (e.g., difficulty simulating human antigen exposure patterns), yet investigating AR’s immunomodulatory effects in HP is clinically valuable--timely inhibition of acute HP progression could reduce the risk of chronic pulmonary fibrosis and improve long-term prognosis.

#### 9.2.4 Methodological limitations in included studies

Most APS-focused studies lacked standardized endotoxin (LPS) testing or decontamination procedures. As LPS is a potent endogenous ligand of TLR4, undetected contamination may interfere with observations related to TLR4/NF-κB signaling, reducing the reliability of mechanistic conclusions. Additionally, few studies reported detailed information on APS purity, structure, or extraction processes, contributing to heterogeneity across findings. Furthermore, while all included animal studies focused on respiratory disease models, not all were established as allergic respiratory disease models--particularly for HP, some mechanistic evidence was derived from pulmonary fibrosis or related respiratory models as alternatives, and extrapolation of these findings to ARDs requires caution to avoid overgeneralization.

In addition, in traditional medical systems other than TCM, such as Indian Ayurveda and Avicenna’s Arabian traditional medicine, relevant textual evidence demonstrates that AR is not officially recorded in the Ayurvedic Pharmacopoeia of India and ancient Ayurvedic classics, and its current application in Ayurveda is a modern introduced practice. Avicenna’s Canon of Medicine only documented tragacanth derived from gum-producing Astragalus species, which named Tragacanth, are congeneric but distinct species from genuine medicinal astragalus. In summary, there is no original classical literature recording the traditional medicinal application of genuine astragalus in either the Ayurvedic or Avicennian medical system.

### 9.3 Future research directions

Addressing the above limitations opens several promising avenues for advancing AR-related research in ARDs:

#### 9.3.1 Strengthen mechanistic exploration of understudied metabolites and pathways

Future studies should focus on undercharacterized metabolites (e.g., AS-II, AS-VII, isorhamnetin, kaempferol, formononetin) and signaling pathways (e.g., JAK2/STAT3), validating their targets and mechanisms through well-designed preclinical experiments. Novel technologies including multi-omics (genomics, proteomics, metabolomics) and single-cell sequencing can uncover unrecognized targets and pathways (e.g., ILC2 regulation, NLRP3-mediated pyroptosis), expanding the mechanistic understanding of AR in ARDs.

#### 9.3.2 Generate high-quality clinical evidence

Well-designed, large-scale, internationally standardized multi-center RCTs of pure AR metabolites (e.g., high-purity AS-IV, APS) are needed to evaluate their safety and efficacy in ARDs, with standardized outcome measures to strengthen clinical validation. Additionally, clarifying potential botanical drug-drug interactions between AR and conventional ARDs therapies (e.g., inhaled corticosteroids, biologics) and assessing safety profiles in special populations (e.g., children, elderly, pregnant women) will enhance clinical applicability.

#### 9.3.3 Expand research on understudied ARD subtypes

Targeted research on AC and HP is essential to address content imbalance, with a focus on developing stable, clinically relevant models to investigate AR’s therapeutic potential. For HP, in particular, validating AR’s immunomodulatory effects could provide critical insights into preventing progression to chronic fibrosis.

#### 9.3.4 Address methodological gaps in APS research

To ensure the reliability of TLR4/NF-κB signaling-related conclusions, future APS studies should prioritize preparations from manufacturers implementing strict endotoxin testing and decontamination controls. For laboratory-purified APS, additional control steps (e.g., LAL assays for endotoxin detection, polymyxin B neutralization, ultrafiltration) should be incorporated to rule out LPS artefacts.

#### 9.3.5 Validate imaging biomarkers for clinical translation

in the context of airway remodeling and pulmonary fibrosis—core pathological features of ARDs—structural airway damage has been widely recognized as a strong prognostic indicator, as it directly correlates with disease progression and long-term clinical outcomes (Phillips et al., 2025; Savin et al., 2023; Richeldi et al., 2017). Integrating this prognostic significance with the well-documented anti-remodeling and anti-fibrotic effects of AR and its bioactive components (e.g., AS-IV, APS, calycosin) provides a promising direction for future clinical translation: identifying imaging biomarkers that can objectively reflect airway structural improvement induced by AR. These imaging biomarkers (e.g., computed tomography [CT]-derived airway wall thickness, fibrotic lesion volume, and lung function parameters; high-resolution CT signs of alveolar structural integrity) could serve as non-invasive, standardized outcome measures to evaluate the therapeutic efficacy of AR-based therapies in ARDs (Phillips et al., 2025). This integration not only strengthens the clinical relevance of AR’s anti-remodeling effects but also facilitates the translation of preclinical findings to clinical practice by providing measurable indicators for therapeutic response.

#### 9.3.6 Optimize bioavailability of AR metabolites

Key metabolites such as AS-IV and flavonoids exhibit poor oral bioavailability due to low solubility and rapid metabolism, limiting clinical translation. Advanced delivery systems (e.g., lipid nanocapsules, polymer micelles) or co-administration with APS (which enhances intestinal mucus permeability and absorption) can improve stability and bioavailability, bridging the gap between effective preclinical doses and expected human exposures.

In conclusion, AR holds significant promise as a TCM suitable for long-term administration in ARDs patients, and its bioactive metabolites represent valuable candidates for the development of novel, targeted therapeutics. Addressing current limitations--including gaps in research on understudied metabolites and ARDs subtypes, insufficient clinical evidence, and methodological issues in APS research—alongside optimizing bioavailability and validating imaging biomarkers, will enable AR to play an increasingly important role in the global management of ARDs.
